# Characteristics of the GlnH and GlnX Signal Transduction Proteins Controlling PknG-Mediated Phosphorylation of OdhI and 2-Oxoglutarate Dehydrogenase Activity in Corynebacterium glutamicum

**DOI:** 10.1128/spectrum.02677-22

**Published:** 2022-11-29

**Authors:** Lea Sundermeyer, Graziella Bosco, Srushti Gujar, Melanie Brocker, Meike Baumgart, Dieter Willbold, Oliver H. Weiergräber, Marco Bellinzoni, Michael Bott

**Affiliations:** a IBG-1: Biotechnology, Institute of Bio- and Geosciences, Forschungszentrum Jülich, Jülich, Germany; b IBI-7: Structural Biochemistry, Institute of Biological Information Processing, Forschungszentrum Jülich, Jülich, Germany; c Institut für Physikalische Biologie, Heinrich-Heine-Universität Düsseldorf, Düsseldorf, Germany; d Institut Pasteur, Université de Paris Cité, CNRS UMR3528, Unité de Microbiologie Structurale, Paris, France; e Bioeconomy Science Center (BioSC), Forschungszentrum Jülich, Jülich, Germany; Centre national de la recherche scientifique, Aix-Marseille Université

**Keywords:** bacterial signaling, *Corynebacterium glutamicum*, 2-oxoglutarate dehydrogenase, protein phosphorylation, periplasmic binding protein, *Actinobacteria*, forkhead-associated domain, four-helix bundle, serine/threonine kinases, signal transduction

## Abstract

In Corynebacterium glutamicum the protein kinase PknG phosphorylates OdhI and thereby abolishes the inhibition of 2-oxoglutarate dehydrogenase activity by unphosphorylated OdhI. Our previous studies suggested that PknG activity is controlled by the periplasmic binding protein GlnH and the transmembrane protein GlnX, because Δ*glnH* and Δ*glnX* mutants showed a growth defect on glutamine similar to that of a Δ*pknG* mutant. We have now confirmed the involvement of GlnH and GlnX in the control of OdhI phosphorylation by analyzing the OdhI phosphorylation status and glutamate secretion in Δ*glnH* and Δ*glnX* mutants and by characterizing Δ*glnX* suppressor mutants. We provide evidence for GlnH being a lipoprotein and show by isothermal titration calorimetry that it binds l-aspartate and l-glutamate with moderate to low affinity, but not l-glutamine, l-asparagine, or 2-oxoglutarate. Based on a structural comparison with GlnH of Mycobacterium tuberculosis, two residues critical for the binding affinity were identified and verified. The predicted GlnX topology with four transmembrane segments and two periplasmic domains was confirmed by PhoA and LacZ fusions. A structural model of GlnX suggested that, with the exception of a poorly ordered N-terminal region, the entire protein is composed of α-helices and small loops or linkers, and it revealed similarities to other bacterial transmembrane receptors. Our results suggest that the GlnH-GlnX-PknG-OdhI-OdhA signal transduction cascade serves to adapt the flux of 2-oxoglutarate between ammonium assimilation via glutamate dehydrogenase and energy generation via the tricarboxylic acid (TCA) cycle to the availability of the amino group donors l-glutamate and l-aspartate in the environment.

**IMPORTANCE** *Actinobacteria* comprise a large number of species playing important roles in biotechnology and medicine, such as Corynebacterium glutamicum, the major industrial amino acid producer, and Mycobacterium tuberculosis, the pathogen causing tuberculosis. Many actinobacteria use a signal transduction process in which the phosphorylation status of OdhI (corynebacteria) or GarA (mycobacteria) regulates the carbon flux at the 2-oxoglutarate node. Inhibition of 2-oxoglutarate dehydrogenase by unphosphorylated OdhI shifts the flux of 2-oxoglutarate from the TCA cycle toward glutamate formation and, thus, ammonium assimilation. Phosphorylation of OdhI/GarA is catalyzed by the protein kinase PknG, whose activity was proposed to be controlled by the periplasmic binding protein GlnH and the transmembrane protein GlnX. In this study, we combined genetic, biochemical, and structural modeling approaches to characterize GlnH and GlnX of C. glutamicum and confirm their roles in the GlnH-GlnX-PknG-OdhI-OdhA signal transduction cascade. These findings are relevant also to other *Actinobacteria* employing a similar control process.

## INTRODUCTION

Corynebacterium glutamicum is a nonpathogenic Gram-positive soil bacterium that is used in large-scale amino acid production (1–4). It belongs to the order *Corynebacteriales* within the *Actinobacteria*, which includes important human pathogens such as Corynebacterium diphtheriae and Mycobacterium tuberculosis (5). Due to the importance of C. glutamicum in industrial biotechnology, its metabolism and regulatory mechanisms have been extensively studied as a prerequisite for strain development by metabolic engineering. We previously identified a novel type of posttranslational regulation controlling the carbon flux at the 2-oxoglutarate metabolic branching point in C. glutamicum (6). At this node, 2-oxoglutarate is either oxidatively decarboxylated to succinyl coenzyme A (succinyl-CoA) by an unusual 2-oxoglutarate dehydrogenase complex (ODH) within the tricarboxylic acid (TCA) cycle (7) or reductively aminated to l-glutamate by glutamate dehydrogenase (GDH). We showed that the activity of ODH is inhibited by the 15-kDa forkhead-associated (FHA) domain-containing protein OdhI. OdhI binds with nM affinity to the OdhA subunit of the ODH and thereby inhibits its activity (6, 8, 9). Phosphorylation of the Thr14 residue of OdhI by the serine/threonine protein kinase PknG leads to a conformational change of OdhI and thereby prevents its binding to OdhA, while the phospho-serine/threonine protein phosphatase Ppp reactivates OdhI by dephosphorylation (6, 8, 10). Not only PknG, but also the serine/threonine protein kinases (STPK) PknA, PknB, and PknL are able to phosphorylate OdhI, at least *in vitro* (10). The conformational change of OdhI upon phosphorylation was demonstrated by structural analysis via nuclear magnetic resonance (NMR) (11). In addition to phosphorylation, succinylation of OdhI at K132 was also reported to diminish the inhibitory effect on ODH (12). The structure of PknG of C. glutamicum has also been elucidated recently and revealed features required for interaction with OdhI and signal transduction (13). In a Δ*pknG* mutant, the internal l-glutamate concentration was increased 2-fold, confirming the importance of PknG in glutamate metabolism (6). The inhibition of ODH activity by OdhI enables a shift of the carbon flux from the TCA cycle toward l-glutamate synthesis and thus nitrogen assimilation. Glutamate overproduction by an *odhI* deletion mutant of C. glutamicum was drastically impaired, revealing that OdhI is crucial for this process (14). This novel type of posttranslational regulation of ODH was shown to be also present in Mycobacterium species, where the OdhI homolog is named GarA (15–19).

For a physiological understanding of the control of the OdhI phosphorylation status and thus of the regulation of carbon flux at the 2-oxoglutarate node, it is crucial to know how the activity of PknG is regulated. In contrast to the other STPKs of C. glutamicum (PknA, PknB, PknL), PknG is a cytoplasmic protein lacking extracellular or transmembrane segments which could act as receptors for extracellular nutrients (10, 13). The *pknG* gene is located in a putative operon with *glnH* and *glnX*, which were initially annotated to encode a putative secreted glutamine binding protein and a membrane protein, respectively (6, 20). Deletion of either *glnH* or *glnX* in C. glutamicum led to a growth defect comparable to that caused by deletion of *pknG* when the strains were grown on glutamine as the sole nitrogen and carbon source (6). This result suggested that GlnH, GlnX, and PknG are part of a signal transduction cascade in which GlnH serves as an extracellular sensor, presumably for amino acids, whereas GlnX transmits the ligandation status of GlnH to PknG to control its kinase activity. In line with such a model, deletion of *glnX* in Mycolicibacterium smegmatis mimicked the growth defect of the *pknG* deletion strain on glutamate, and the GlnH proteins of Mycobacterium tuberculosis and C. glutamicum were shown to bind l-aspartate and l-glutamate (21). Remarkably, the affinities of M. tuberculosis GlnH for these amino acids were about 100-fold higher than those of C. glutamicum GlnH (21).

In this study, we confirmed the involvement of GlnH and GlnX in signal transduction to OdhI and ODH in C. glutamicum by analyzing the influence of Δ*glnH* and Δ*glnX* mutants on the OdhI phosphorylation status and glutamate overproduction and by exploring the genetic alterations of Δ*glnX* suppressor mutants that regained the ability to grow on glutamine agar plates. We analyzed whether GlnH is a lipoprotein, determined its ligand binding properties, and identified two residues critical for the ligand binding affinity. Finally, we analyzed the membrane topology of GlnX and predicted its overall architecture and functional properties based on a structural model.

## RESULTS

### Complementation of the growth defect of the Δ*glnX2* and Δ*glnH* mutants of C. glutamicum on glutamine agar plates.

In our previous study we reported that Δ*glnX* and Δ*glnH* mutants of C. glutamicum show the same growth defect on glutamine agar plates as the Δ*pknG* mutant (6). The growth defect of the Δ*glnH* mutant could be complemented by transformation with the plasmid pAN6-*glnH* expressing *glnH* under the control of the *tac* promoter (see Fig. S1 in the supplemental material). As initial complementation experiments with the Δ*glnX* mutant failed, we constructed another deletion mutant, Δ*glnX2*, in which 59 5′-terminal *glnX* codons and 142 3′-terminal *glnX* codons were retained and the 301 codons in between were deleted and replaced by an artificial 21-bp sequence. The Δ*glnX2* mutant showed the expected growth defect on glutamine agar plates and could be complemented by transformation with the plasmid pJC1-*glnX*Prom expressing *glnX* from its native promoter (Fig. S1). These results show that the growth defect on glutamine agar plates of the Δ*glnX2* and Δ*glnH* mutants is not caused by polar effects on *pknG* expression. The observation that the original Δ*glnX* mutant could not be complemented might be due to the presence of a yet unidentified promoter within the 3′-end of the *glnX* coding region that was retained in the Δ*glnX2* mutant.

### Influence of *glnH* and *glnX* deletions on the phosphorylation status of OdhI and glutamate secretion.

**FIG 1:**
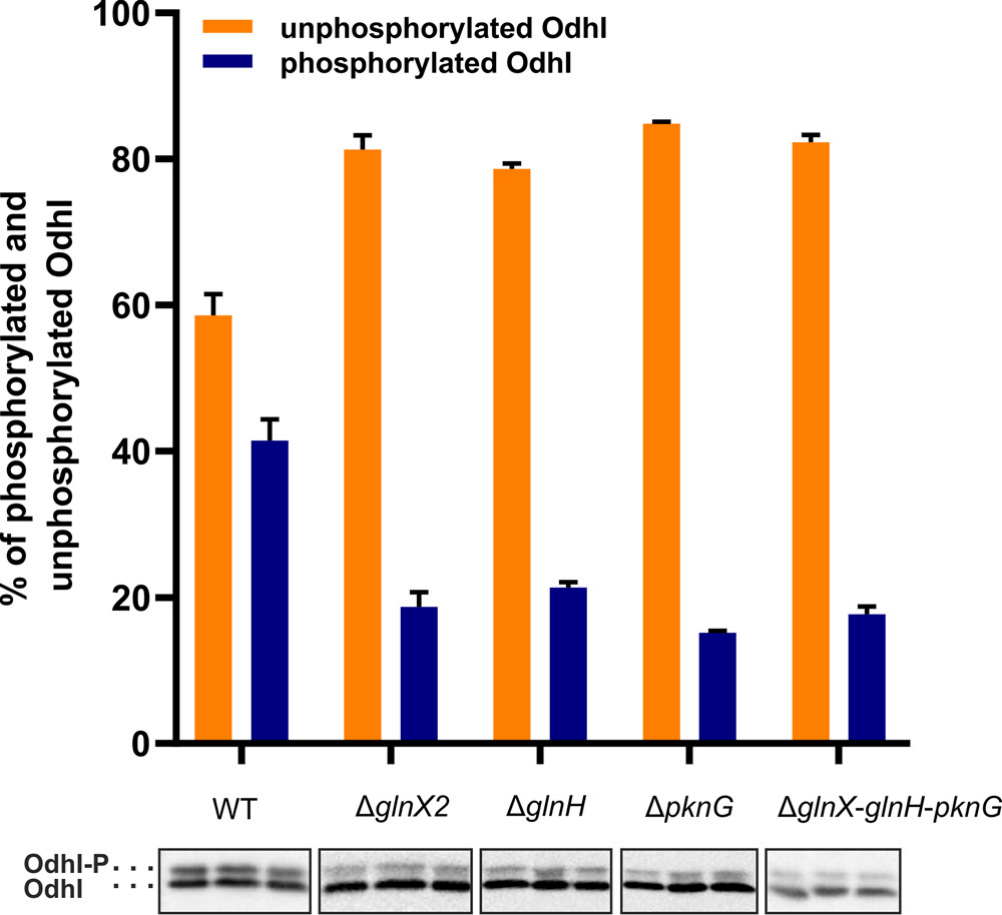
Analysis of the OdhI phosphorylation status in cells grown for 24 h in BHI medium with 4% (wt/vol) glucose. Western blot analysis of cell-free protein extracts (5 μg each) of the indicated C. glutamicum strains was performed with polyclonal anti-OdhI antibodies. The experiment was performed in triplicate. The upper band in the Western blot represents singly or doubly phosphorylated OdhI; the lower band represents unphosphorylated OdhI (14). The percentages (mean values with standard deviation) of unphosphorylated OdhI (orange bars) to phosphorylated OdhI (blue bars) were calculated by densitometry.

We previously showed that the lack of PknG resulted in a reduced level of phosphorylated OdhI during growth in BHI medium with glucose (10). To test how the absence of GlnH or GlnX influences the OdhI phosphorylation status under these conditions, the C. glutamicum strains wild type (WT), Δ*glnX2*, Δ*glnH*, Δ*pknG*, and Δ*glnX-glnH-pknG* were grown overnight in brain heart infusion (BHI) medium with 4% (wt/vol) glucose, and cell-free protein extracts were then analyzed in Western blots with anti-OdhI antiserum. As shown in Fig. 1, ~40% of OdhI was phosphorylated in the WT, but only between 15% and 20% was phosphorylated in the four mutants. This result indicated that the absence of GlnH and GlnX influences the phosphorylation status of OdhI in a similar manner and extent as the absence of PknG. The simultaneous absence of all three proteins (GlnH, GlnX, and PknG) in the Δ*glnX-glnH-pknG* mutant had the same effect on OdhI phosphorylation as the three individual gene deletions, which is expected if each of the three proteins mediates a distinct and essential step of the same signal transduction cascade.

A reduced phosphorylation of OdhI is expected to cause a strengthened inhibition of ODH activity by unphosphorylated OdhI. In agreement with this assumption, the deletion of *pknG* was previously shown to result in increased glutamate excretion compared to the WT, when cells were cultured in the presence of ethambutol, an inhibitor of cell wall arabinogalactan synthesis causing alterations of the cell wall (14). Ethambutol was shown to trigger glutamate secretion by C. glutamicum (22). We now tested the mutants Δ*glnX2* and Δ*glnH* regarding glutamate excretion in the presence of ethambutol and observed a 2-fold-increased glutamate titer in the supernatant compared to the WT (Fig. S2), supporting an increased flux of 2-oxoglutarate to l-glutamate due to an enhanced level of unphosphorylated OdhI. Increased glutamate production could be abolished by plasmid-based expression of *glnX* or *glnH* in the respective deletion mutants (Fig. S2).

### Analysis of suppressor mutants selected by growth of strains Δ*glnH* and Δ*glnX2* on glutamine agar plates.

**FIG 2:**
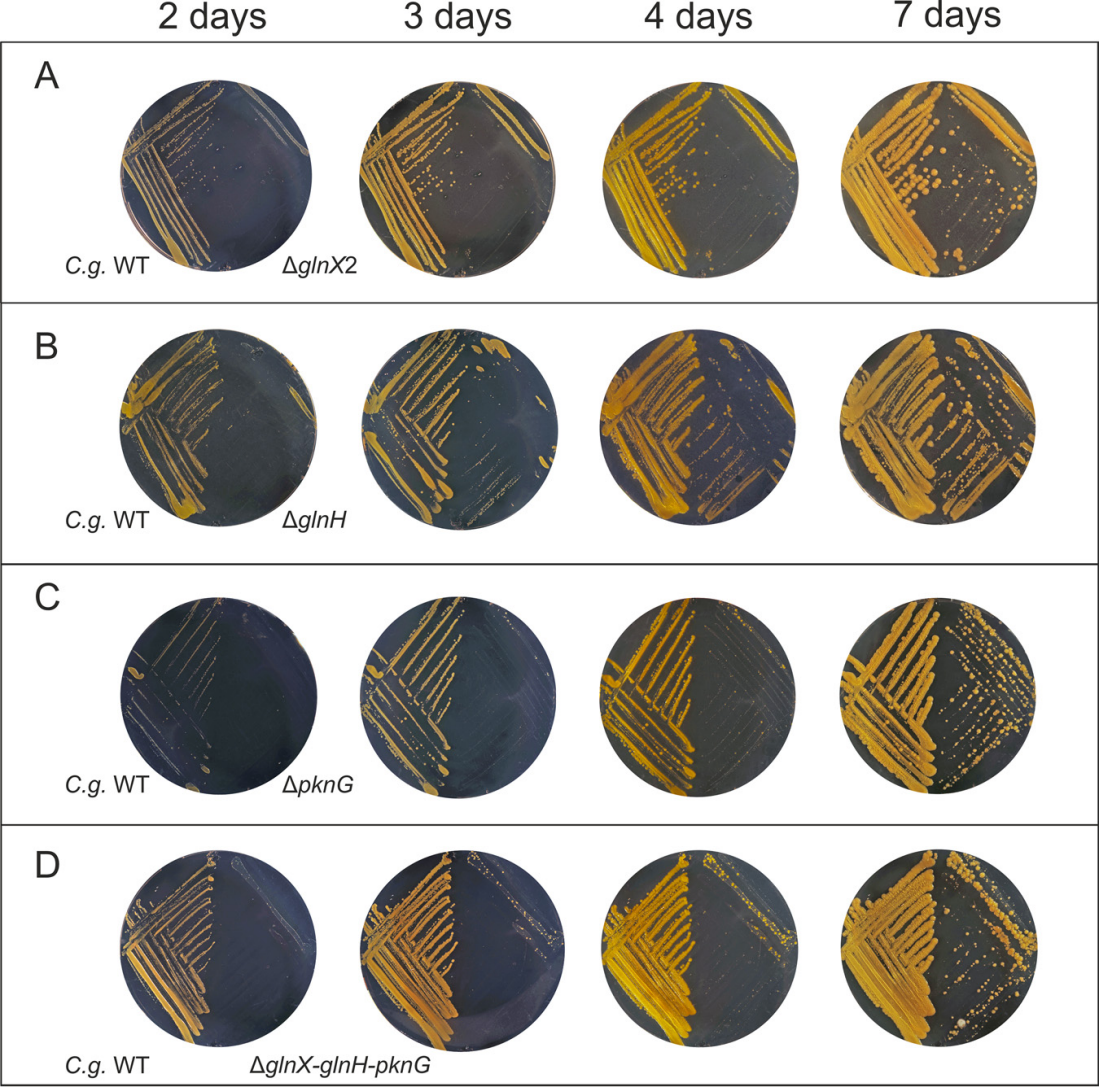
Growth of C. glutamicum WT and the indicated mutant strains on agar plates containing 100 mM l-glutamine as the sole carbon and nitrogen source. The plates were incubated at 30°C for 7 days and photographed after 2, 3, 4, and 7 days.

Prolonged incubation of the Δ*pknG* strain on glutamine agar plates led to the formation of suppressor mutants that regained the ability to grow on glutamine (9). To check for a similar behavior in the case of *glnX* or *glnH* deletions, C. glutamicum WT and the mutant strains Δ*glnX2*, Δ*glnH*, Δ*pknG*, and Δ*glnX-glnH-pknG* were cultivated on glutamine agar plates and photographed after 2, 3, 4, and 7 days (Fig. 2). All mutants showed only marginal growth after 2 days, whereas the WT grew well. During further incubation, all mutants formed colonies with a very heterogeneous size, suggesting the occurrence of suppressor mutations enabling growth.

To test whether mutations in *odhI* are responsible for abolishing the growth defect on glutamine, we sequenced the *odhI* gene of 20 Δ*glnX2* suppressor mutants. As shown in Table 1, 16 of the 20 suppressor mutants (named K1 to K20) in fact contained mutations in *odhI*. In five mutants a stop codon was introduced at five different positions (residue 60 in mutant K2, residue 129 in mutant K3, residue 53 in mutant K5, residue 65 in mutant K17, and residue 123 in mutant K20). In three cases, frameshift mutations were found in codon 59 (mutant K11, 1-bp insertion) or in codon 120 (mutants K12 and K19, 1-bp deletion). In seven cases, mutations leading to a single amino acid exchange were identified: R87P in mutants K1 and K4, E91K in mutants K6 and K16, L107V in mutant K8, F137S in mutant K9, and T84I in mutant K10. In the case of mutant K7, two amino acid exchanges were identified, R87P and E91K. In four suppressor mutants (K13, K14, K15, K18) no mutation was found in *odhI*. We therefore also sequenced *odhA* and *pknG* in these four strains but could not find mutations in these genes.

**TABLE 1:** Overview of mutations identified in the *odhI* gene^*a*^

Mutant	Mutation in *odhI*	Position in *odhI* ORF^*b*^	Codon exchange	Effect on amino acid sequence
K1	GTTTCACGTCGCCAC	260	CGT → CCT	R87P
K2	GGCGCTCGATTCCTT	178	CGA → TGA	R60Stop
K3	GAGATCCAGATTGGC	385	CAG → TAG	Q129Stop
K4	GTTTCACGTCGCCAC	260	CGT → CCT	R87P
K5	CTGGACCAGCCAACC	195	CAG → TAG	Q53Stop
K6	ATGGAGTCGAGCACC	84	TCG → TCA	Silent (S28)
	CACGCAGAGTTCCGC	271	GAG → AAG	E91K
	GTCATGCAGACCGGT	369	CAG → CAA	Silent (Q123)
	GATGAGATCCAGATT	381	GAG → GAA	Silent (E127)
		387	CAG → CAA	Silent (Q129)
K7	GTTTCACGTCGCCAC	260	CGT → CCT	R87P
	CACGCAGAGTTCCGC	271	GAG → AAG	E91K
K8	GGGTCCCTCAACGGA	319	CTC → GTC	L107V
K9	CTGGTTTTCCTCGCA	410	TTC → TCC	F137S
K10	GATGTCACCGTTTCA	251	ACC → ATC	T84I
K11	GCAGGCGCTCGATTC	176	GCT → GCAT	Frameshift after A59
K12	AACGCTCAGGTCATG		CAG → C-G	Frameshift after A119
K13	No mutation in *odhI*			
K14	No mutation in *odhI*			
K15	No mutation in *odhI*			
K16	CACGCAGAGTTCCGC	271	GAG → AAG	E91K
K17	ATGGAGTCGAGCACC	84	TCG → TCC	Silent (S28)
	GCAGGCGCTCGATTC	176	GCT → GAT	A59D
	CTGGACCAGCCAACC	193	CAG → TAG	Q65Stop
K18	No mutation in *odhI*			
K19	AACGCTCAGGTCATG	359	CAG → C-G	Frameshift after A119
K20	GTCATGCAGACCGGT	367	CAG → TAG	Q123Stop

a 20 C. glutamicum Δ*glnX2* suppressor mutants (K1 to K20) forming colonies on glutamine agar plates.

b ORF, open reading frame.

**FIG 3:**
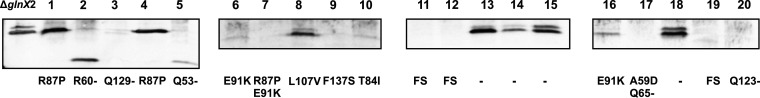
Western blot analysis with anti-OdhI antiserum using crude extracts from C. glutamicum Δ*glnX2* as the positive control and 20 different Δ*glnX2* suppressor mutants (K1 to K20) that were able to grow on glutamine agar plates again. The strains were cultivated in BHI medium with 4% (wt/vol) glucose. In each case, 10 μg of protein was separated by SDS-PAGE (15% separating gel) and used for Western blotting. On the bottom line, the OdhI mutations identified in the 20 Δ*glnX2* suppressor mutants are listed (R60-, Q53-, Q65-, Q123-, nonsense mutations; FS, frameshift mutations; –, no mutation found in *odhI*, *odhA*, and *pknG*).

We assumed that the 16 mutated *odhI* genes led to OdhI variants whose ability to inhibit ODH activity was impaired or abolished. To test if the OdhI protein was still formed in the mutant strains, Western blot analysis was performed with an anti-OdhI antiserum. As shown in Fig. 3, in many suppressor mutants OdhI of native size was no longer detectable. This holds true for all three variants with frameshift mutations and the nonsense mutations Q123Stop and Q129Stop. In the case of the mutations Q53Stop and R60Stop, truncated variants were detectable. In the four suppressor mutants without an *odhI* mutation, the OdhI protein was detectable in both the unphosphorylated and phosphorylated forms (lower and upper band, respectively), as in the parental strain. In the case of the OdhI variant with the mutation F137S and in the variant bearing the two mutations, R87P and E91K, OdhI was no longer detectable. The OdhI variants with the single-amino acid exchanges R87P and L107V were easily detectable, whereas for the variants with the single mutations T84I and E91K, only a very weak signal was observed, suggesting that the latter two OdhI variants were instable and largely degraded. The results obtained for the Δ*glnX2* suppressor mutants confirm that GlnX is part of the signal transduction cascade controlling OdhI phosphorylation. Further support was obtained by construction of a Δ*glnX2* Δ*odhI* mutant, which was able to grow on glutamine agar plates (Fig. S3).

### Influence of R87P and R87A amino acid exchanges in OdhI on the inhibition of ODH activity.

**FIG 4:**
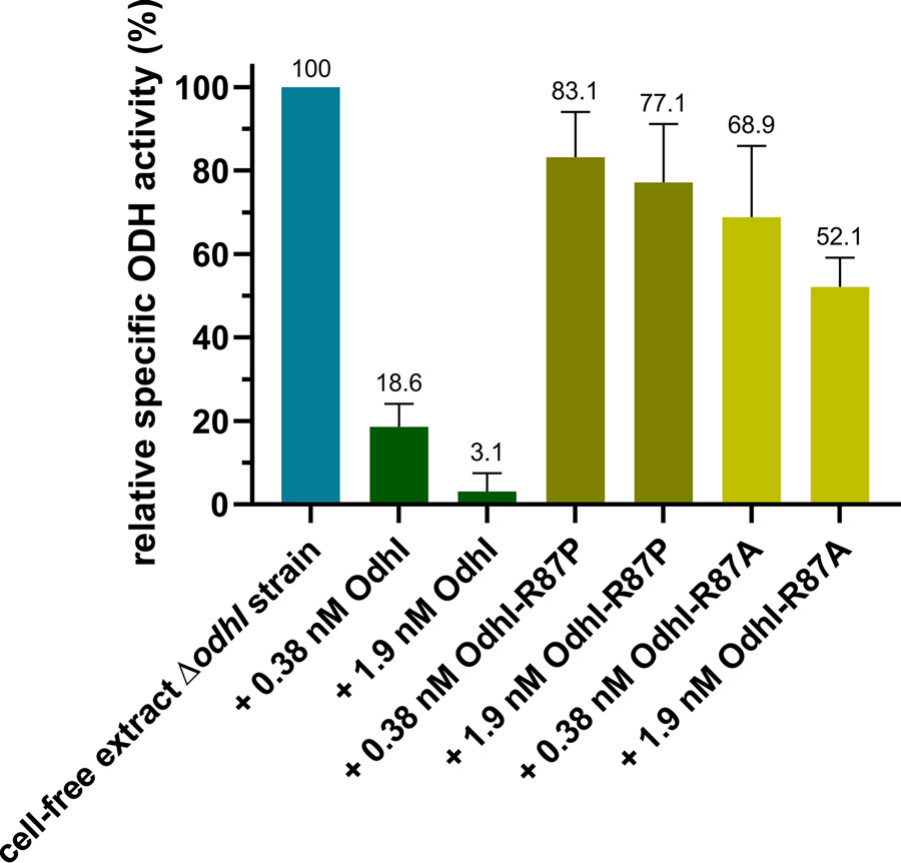
Influence of purified OdhI, OdhI-R87P, and OdhI-R87A on the ODH activity of cell-free extracts of C. glutamicum Δ*odhI*. The activity of the cell extract in the absence of added OdhI was 135 ± 12 nmol min^−1^ (mg protein)^−1^, which was set as 100%. Mean values and standard deviations of three replicates are shown.

In the suppressor mutants with no or barely detectable OdhI or with a truncated OdhI protein, inhibition of ODH activity is likely no longer possible. In the suppressor mutants, in which mutated OdhI variants were still detectable, the amino acid exchanges might have reduced the binding affinity to OdhA and/or the inhibitory effect of OdhI on ODH activity. As the amino acid exchange R87P occurred more than once in the investigated suppressor mutants, we analyzed the influence of this mutation on the inhibition of ODH activity. For this purpose, the proteins OdhI, OdhI-R87P, and OdhI-R87A were overproduced in Escherichia coli and purified (Fig. S4). Their influence on ODH activity was tested with cell-free extracts of C. glutamicum Δ*odhI* as previously described (8). As shown in Fig. 4, wild-type OdhI strongly inhibited ODH activity, with a residual activity of only 3% in the presence of 1.9 nM OdhI, which corresponds to previous results (8). OdhI-R87A and OdhI-R87P at equivalent concentrations also led to an inhibition of ODH activity, but it was much weaker. Even at a concentration of 1.9 nM OdhI-R87A or OdhI-R87P, a residual ODH activity of more than 50% was measured. This suggests that OdhI-R87A and OdhI-R87P are still able to interact with OdhA, but the inhibitory effect is strongly reduced, which might be sufficient to enable growth of the suppressor mutants K1 and K4 on glutamine.

We analyzed the residues OdhI-R87 and OdhI-L107 using an AlphaFold2 model of the OdhI:OdhA complex (Fig. S5), limited to the OdhA E1o domain (residues 365 to 1221). As expected, the model is consistent with the published structure of the mycobacterial KGD:GarA complex (PDB code 6I2Q), with the apical FHA surface of OdhI involved in a number of interactions with OdhA (Fig. S5A). Among these, the conserved residue S86 (OdhI numbering) is predicted to be at a suitable distance to make a hydrogen bond to OdhA-D795 (Fig. S5B), an interaction observed in the KGD:GarA complex, where KGD-D795 was proposed to mimic a phosphorylated threonine, the usual interaction partner of FHA domains (19). According to the AlphaFold2 model, OdhI-R87 is predicted to make a salt bridge with OdhA-D798 from the same long, outer αE helix (Fig. S5B) shown to be, in mycobacterial KGD, one of the main structural elements interacting with GarA; the lack of movement of this helix, hindered as a consequence of GarA binding, locks the enzyme in the resting state (19). The observed substitutions of OdhI-R87 would therefore be expected to remove the predicted salt bridge, leading to a significant decrease in the OdhI binding affinity. In addition, the model predicts OdhI-L107 to be also situated at the FHA surface, where it would be involved in hydrophobic interactions with the N-terminal side of αE (Fig. S5B), mostly with the side chains of A791 and V792. Substitutions of OdhI-L107 may therefore also have a detrimental effect on the OdhI binding affinity and could therefore contribute to relieve the consequences of the accumulation of unphosphorylated OdhI in the suppressor mutants.

### Characterization of GlnH as a lipoprotein.

The GlnH protein is predicted to be a lipoprotein based on the presence of the lipobox LLASCT at the end of the signal peptide (MHAFRRPPPLTTRVGAALLAATLLASCTPT) (23). The predicted cleavage site is between amino acid residues S26 and C27. The thiol group of C27 is presumably modified by the prolipoprotein diacylglyceryl transferase Lgt (Cg2292) to form a diacylglyceryl prolipoprotein with a thioether linkage. The signal peptide is then cleaved by the prolipoprotein signal peptidase LspA (Cg2347), thereby liberating the α-amino group of C27. Previous studies with the lipoprotein AmyE (Cg2705) have shown that the α-amino group of the N-terminal cysteine is acylated by apolipoprotein-*N*-acyltransferase Ppm2 (Cg1673) to form triacylated AmyE (24), and a similar process can be envisaged for GlnH.

**FIG 5:**
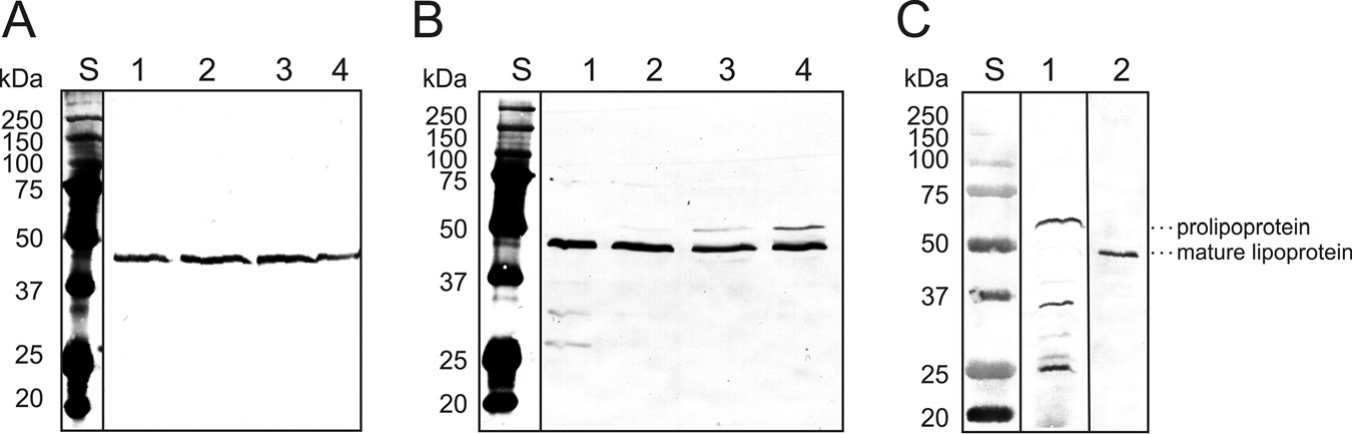
Characterization of GlnH as a lipoprotein. (A to C) Western blot analysis of crude extracts of C. glutamicum Δ*glnH*/pAN6-*glnH* (A and B, lanes 1 to 4; C, lane 2) and C. glutamicum Δ*glnH*/pAN6-*glnH*-C27A (C, lane 1). The plasmid-encoded GlnH contains a C-terminal StrepTag-II, which was detected by Streptactin-alkaline phosphatase conjugate. Panel A shows the control samples without globomycin, and panel B shows the samples where globomycin was added during exponential growth. Samples were taken immediately before (A and B, lane 1) and 1 h, 2 h, and 3 h after globomycin addition (A and B lanes 2, 3, and 4). In panel C, a comparison of the GlnH-C27A variant (lane 1) and wild-type GlnH (lane 2) is shown. Mutated GlnH-C27A cannot be modified by prolipoprotein diacylglycerol transferase or cleaved by the lipoprotein signal peptidase. In panels A and B, 10 μg protein was applied per lane. In panel C, 50 μg protein of crude extract was applied in lane 1 and 5 μg membrane protein was applied in lane 2. Lane S shows the molecular mass standards. The lanes in panels A, B, and C were derived from a single blot in each case and arranged for better visibility of the relevant features.

To confirm that GlnH is a lipoprotein, the cyclic peptide antibiotic globomycin was used, which specifically inhibits the activity of the lipoprotein signal peptidase LspA and thus the cleavage of the signal peptide (25–27). C. glutamicum Δ*glnH*/pAN6-*glnH*, which encodes a C-terminally Strep-tagged GlnH, was cultivated in CGXII-glucose medium supplemented with 1 mM IPTG (isopropyl-β-d-thiogalactopyranoside) and 50 μg/mL globomycin to prevent lipoprotein maturation. Crude extracts of samples taken before and hourly after globomycin addition were analyzed by Western blot analysis with StrepTactin-alkaline phosphatase conjugate. As shown in Fig. 5, in the control without globomycin (Fig. 5A), only the mature GlnH protein with an apparent mass of about 45 kDa (predicted mass of about 36 kDa) was visible, whereas in the samples treated with globomycin (Fig. 5B), a GlnH variant with a larger apparent mass became increasingly visible. This enlarged variant presumably represents the diacylglyceryl prolipoprotein form of GlnH still containing the signal peptide. Further support for this interpretation was obtained by Western blot analysis of the strain C. glutamicum Δ*glnH*/pAN6-*glnH*-C27A, in which C27 was changed to alanine, thereby preventing thiol modification of C27 and cleavage of the signal peptide. In this case, only the GlnH prolipoprotein was detected (Fig. 5C).

### Characterization of the ligand-binding properties of GlnH.

**FIG 6:**
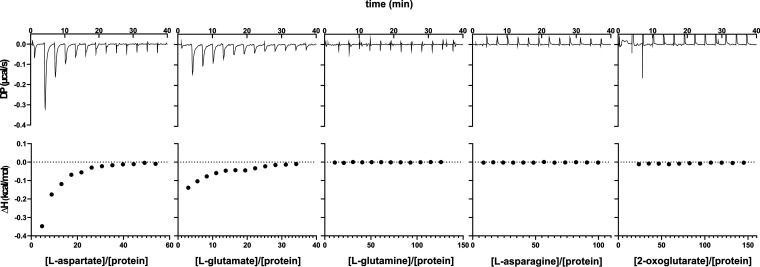
Representative ITC experiments with His_10_-GlnHΔSP and the indicated ligands. The experiments were performed in 40 mM HEPES buffer, pH 7, with 100 mM KCl and 10 mM MgCl_2_.

Based on the similarity to the glutamine-binding protein GlnH of E. coli, which is part of the ABC transporter GlnHPQ (28, 29), the protein encoded by cg3045 in the C. glutamicum genome was initially annotated as a putative glutamine binding protein (20). While the phenotype of the Δ*glnH* mutant provided clear evidence for a role of GlnH in the glutamine metabolism of C. glutamicum, the glutamine uptake activity of a Δ*glnH* mutant (and a Δ*glnX* mutant) was not strongly affected, suggesting that GlnH might not be involved in glutamine uptake (6). To characterize the ligand binding properties of GlnH, a soluble protein version lacking the N-terminal signal peptide and the lipobox motif was overproduced in E. coli BL21(DE3) using the expression plasmid pET-TEV-*glnH*ΔSP and purified by Ni^2+^-chelate affinity chromatography. The His-tagged protein was used for interaction studies with different potential ligands using isothermal titration calorimetry (ITC). Based on the annotation, l-glutamine was first tested as a ligand, but no binding to His_10_-GlnHΔSP was observed under the conditions used (40 mM HEPES, pH 7, 100 mM KCl, 10 mM MgCl_2_). In contrast, l-aspartate and l-glutamate served as ligands for His_10_-GlnHΔSP, although with moderate affinity. From five independent experiments each, a mean equilibrium dissociation constant (*K_D_*) value of 264 ± 14.6 μM was obtained for l-aspartate, whereas for l-glutamate the affinity was about five times lower with a mean *K_D_* of 1,256 ± 220 μM. In both cases, binding was exothermic with mean observed enthalpy changes of −18.5 ± 4.3 kJ/mol for l-aspartate and −17.5 ± 5.0 kJ/mol for l-glutamate. Besides l-glutamine, l-aspartate, and l-glutamate, l-asparagine and 2-oxoglutarate were also tested as potential ligands, but no interaction with His_10_-GlnHΔSP was observed. In Fig. 6, representative ITC experiments are shown for the five ligands tested.

### Analysis of structural differences between the GlnH proteins of M. tuberculosis and C. glutamicum.

**FIG 7:**
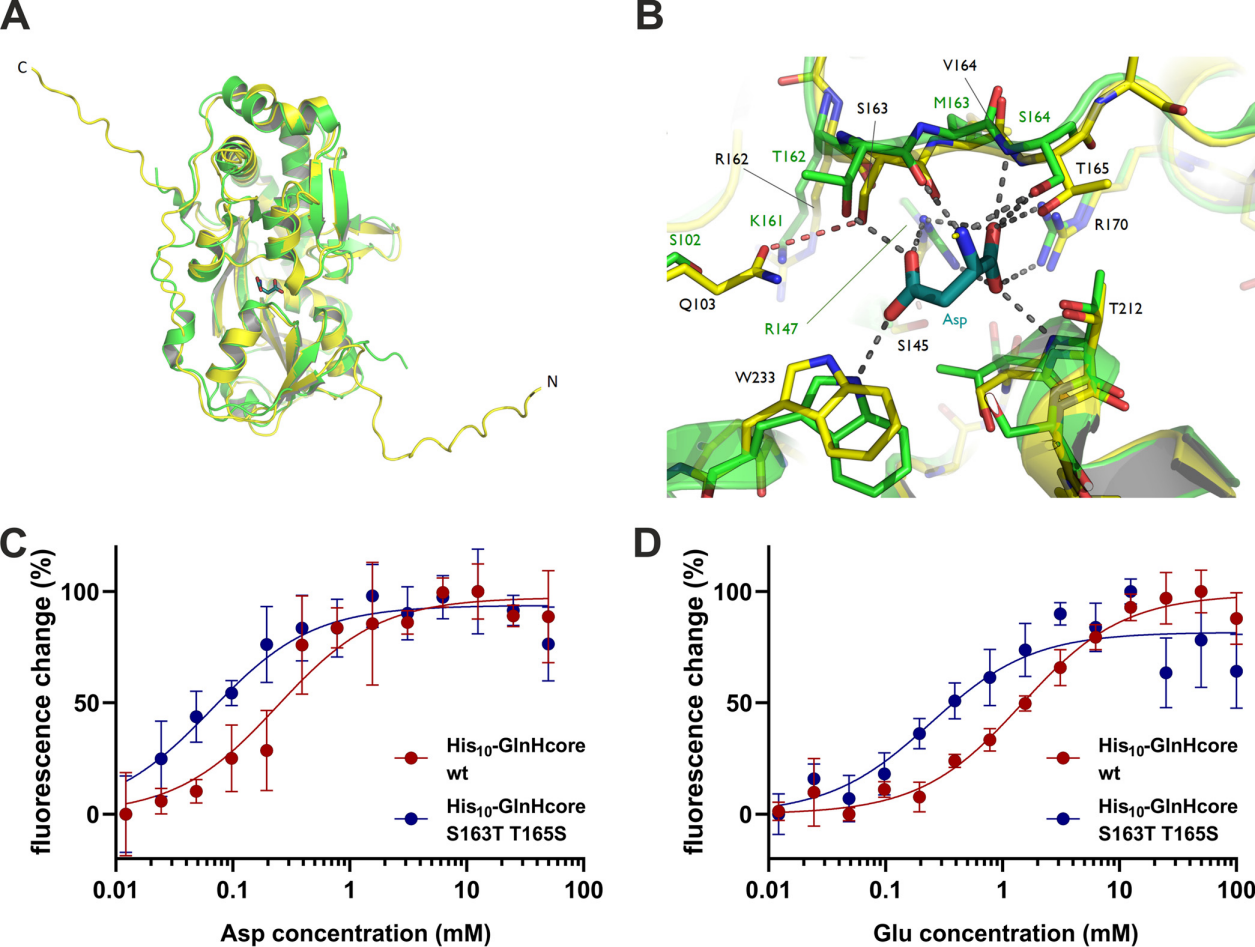
(A) Superimposition of the AlphaFold2 model for GlnHΔSP (yellow) on the crystal structure of M. tuberculosis GlnH in complex with aspartate (green; PDB code 6H1U). Both the N- and C-terminal ends of GlnHΔSP are predicted to be unstructured (residues 27 to 50 and 334 to 344, respectively; the N- and C-terminal ends are indicated). (B) Zoomed view of the ligand binding site showing residues involved in ligand binding in the M. tuberculosis GlnH-aspartate complex (gray dashed lines represent H-bond interatomic distances) and the equivalent positions in the C. glutamicum GlnHΔSP model (yellow; dashed lines in salmon color indicate potential H-bonds specific for C. glutamicum GlnH). (C and D) Influence of S163T-T165S swap in C. glutamicum GlnH on the ligand binding affinity for l-aspartate and l-glutamate. Ligand binding was analyzed measuring the intrinsic tryptophan fluorescence (excitation 292 nm; emission 340 nm) at different sodium aspartate and sodium glutamate concentrations. Dissociation constants were calculated using a one-site specific binding fit. Shown are mean values of 5 to 6 replicates. Error bars represent the standard deviation.

A previous study, focused on the orthologous GlnH protein from M. tuberculosis, reported 50- to 135-fold lower *K_D_* values for l-aspartate (4.8 ± 0.6 μM) and l-glutamate (15.2 ± 5.7 μM) compared to C. glutamicum GlnH and provided structural evidence of ligand binding to GlnH through the crystal structures of the M. tuberculosis protein in complex with either l-aspartate, l-glutamate, or l-asparagine (21). To get insights into GlnH ligand binding specificity and the structural features underlying the different affinities of the two orthologues, we generated a structural model for C. glutamicum GlnHΔSP using AlphaFold2 (30). The models superimpose on the experimental structure of M. tuberculosis GlnH in complex with l-aspartate (PDB code 6H1U) with a root-mean-square distance (RMSD) of around 0.8 Å (Fig. 7A), indicating that, despite the nonnegligible differences in their amino acid sequences (43% sequence identity), the ligand binding domains of the two orthologues are not expected to show significant differences in their overall fold. The comparison of the ligand binding pockets between the two proteins shows, however, a few subtle differences. Specifically, residues in the loop 160 to 166 (C. glutamicum GlnH numbering, including signal peptide), which is crucial for ligand binding, are not fully conserved. Most notably, two nearby residues (Thr162 and Ser164 in M. tuberculosis), whose hydroxyl groups act as hydrogen bond (H-bond) donors toward the ligand carboxyl groups, have their relative positions swapped in C. glutamicum, where they are replaced by Ser163 and Thr165, respectively (Fig. 7B). Also, the Ser163 hydroxyl group potentially falls within H-bond distance from the amide group of Gln103 (Ser102 in M. tuberculosis), which, in turn, may contribute to making it unavailable as an H-bond donor to the incoming amino acid ligand (Fig. 7B).

To test the relevance of the amino acid residues S163 and T165 for ligand binding affinity, a GlnH variant (His_10_-GlnHcore-S163T-T165S) with the amino acid exchanges S163T-T165S was isolated, and its ligand binding properties were compared with the parental protein by measuring the intrinsic tryptophan fluorescence at various ligand concentrations (Fig. 7C and D). For the parental His_10_-GlnHcore protein, *K_D_* values of 242 μM for l-aspartate and 1,458 μM for l-glutamate were determined, which are comparable to the *K_D_* values determined by ITC. For the His_10_-GlnHcore-S163T-T165S protein, *K_D_* values of 65 μM for l-aspartate and 243 μM for l-glutamate were measured. The 4- to 6-fold-increased binding affinity caused by a swap of S163 and T165 is remarkable and confirms the relevance of these residues for the ligand binding properties of GlnH.

### Analysis of the GlnX topology using PhoA and LacZ fusions.

**FIG 8:**
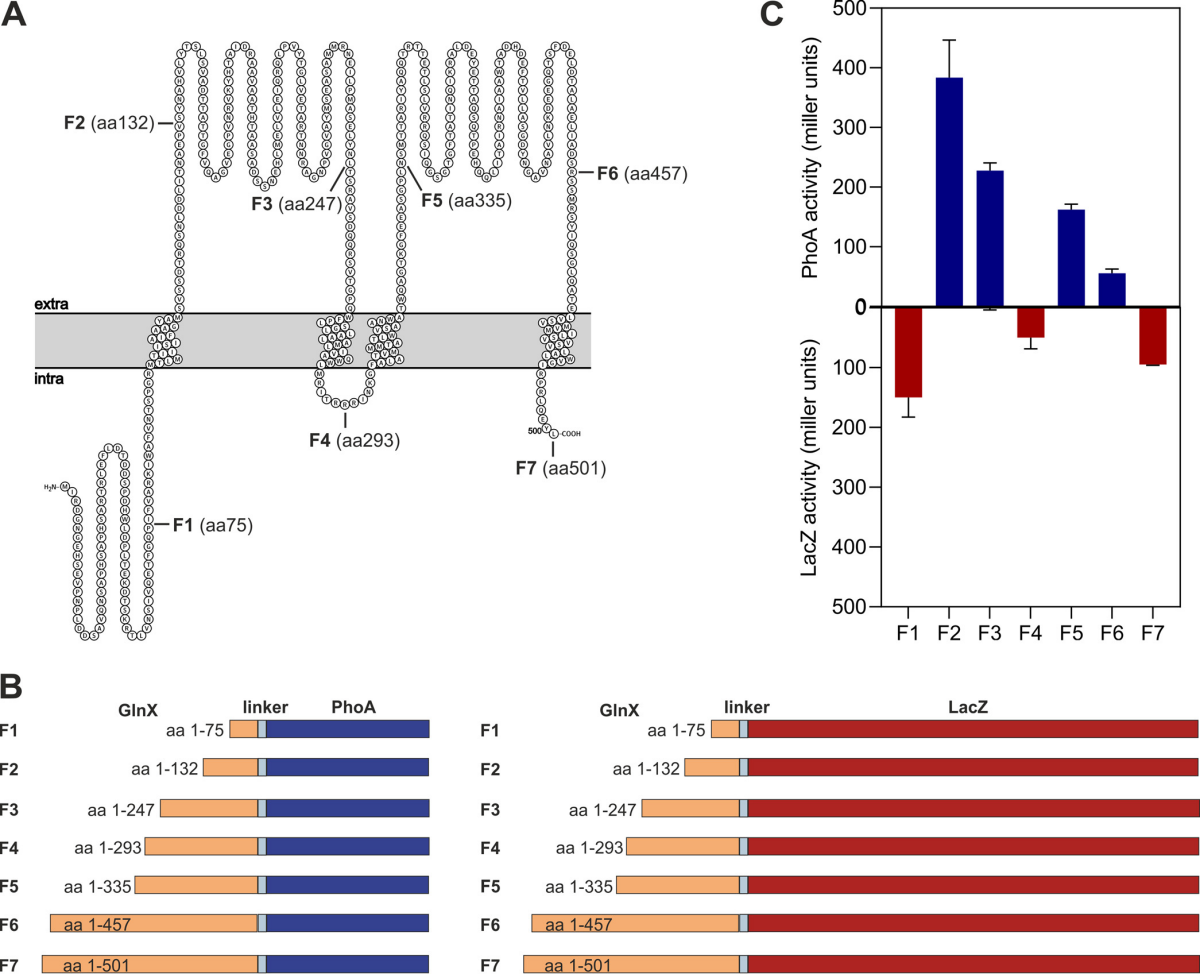
Analysis of the GlnX topology. (A) Predicted topology of GlnX using PROTTER software (37). The seven positions selected for creating PhoA and LacZ fusions are indicated. (B) Scheme of GlnX-PhoA and GlnX-LacZ fusion proteins. (C) PhoA and LacZ activities determined with E. coli TG1 cells carrying pPREx2-*glnX*-*phoA*-F1 to -F7 and pPREx2-*glnX*-*lacZ*-F1 to -F7 expression plasmids. Cells were cultivated at 30°C in LB medium, and gene expression was induced for 1 h by addition of 1 mM IPTG in the exponential growth phase. Subsequently, cells were permeabilized, and PhoA and LacZ activities were determined using the artificial substrates *p*-nitrophenylphosphate and *o*-nitrophenyl-galactopyranoside, respectively, by measuring the absorbance at 420 nm. Shown are mean values and standard deviation of biological triplicates.

Bioinformatic analysis of the GlnX sequence resulted in the topology shown in Fig. 8A. We based our analyses on a GlnX protein composed of 501 amino acid residues with the N-terminal sequence MIRDGNGEH, assuming a leaderless mRNA formed from the reported transcriptional start site of *glnX* (31), which was confirmed by us via 5′-rapid amplification of cDNA ends (RACE) experiments (data not shown). The GlnX protein is predicted to contain four transmembrane helices (TMHs) and two periplasmic domains comprising approximately 156 and 153 amino acid residues. Three portions of the protein are predicted to face the cytoplasm, which are the N-terminal region comprising about 110 amino acid residues, a small portion of about 11 residues linking TMH-2 and TMH-3, and the C-terminal region of about eight amino acid residues. A search for sequence signatures using the Pfam database matched the second periplasmic region of GlnX to the CHASE3 family, which represents extracellular sensory domains found in various classes of bacterial transmembrane proteins, including histidine kinases and chemoreceptors (32).

To experimentally confirm the predicted topology, seven fusion proteins combining different portions of GlnX with either alkaline phosphatase (PhoA) or β-galactosidase (LacZ) of E. coli were constructed (Fig. 8B). While PhoA is active only if located in the periplasm, LacZ shows activity only when located in the cytoplasm (33, 34). Three fusion sites were positioned in predicted cytoplasmic regions (residues 75, 293, and 501), whereas four fusion sites were located in the two predicted periplasmic regions (residues 132, 247, 335, and 457). The plasmids for expression of the 14 fusion genes were constructed in the vector pPREx2 (35), and E. coli TG1 served as the host for the PhoA and LacZ activity measurements. As shown in Fig. 8C, the results confirmed the predicted topology. The fusions at position 75, 293, and 501 showed LacZ, but no PhoA activity, whereas the fusions at positions 132, 247, 335, and 457 possessed PhoA, but no LacZ activity.

### Structural model of the GlnX protein.

**FIG 9:**
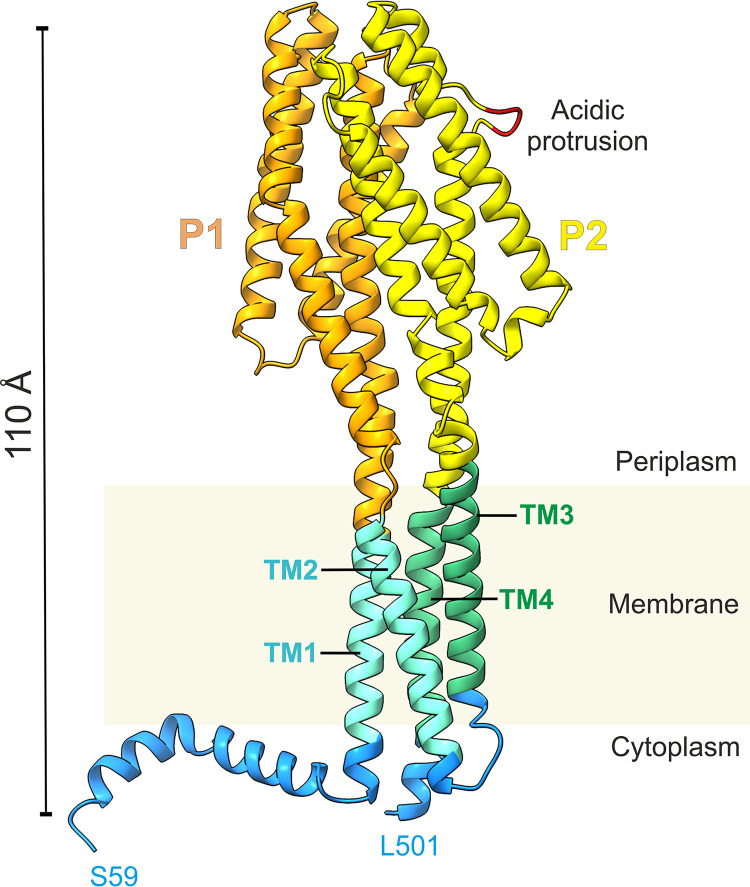
Structural model of GlnX in ribbon representation. The model is colored according to the topology prediction by DeepTMHMM (66) for the periplasmic, cytoplasmic, and transmembrane (TM) segments (orange, P1; yellow, P2; cyan, TM1 and TM2; green, TM3 and TM4; blue, cytoplasmic parts). Residues ^435^DEE^437^ in the acidic H4′ protrusion (highlighted in red) are hypothesized to be involved in interactions with GlnX ligands (see the text for more details). The helix preceding TM1 displays significant amphipathic character and could be preferentially anchored to the membrane. The N-terminal residues 1 to 58 are not shown in this model, as they were predicted to be disordered.

AlphaFold2 was used to predict the structure of the GlnX protein. The *ab initio* prediction was executed using parameters described in Materials and Methods. The run produced five models ranked according to the model confidence level, i.e., the predicted local distance difference test (pLDDT) (Fig. S6). The individual models displayed a similar overall architecture, with high to moderate model confidence in most regions; for description, we will use the top-scoring model from the first run (Fig. 9). As anticipated from conventional secondary structure predictions, the AlphaFold2 model of GlnX features a very high α-helical content, and its membrane topology is in accordance with our own prediction and experimental evidence. Specifically, 79% of all residues are involved in either α-helices, 3_10_-helices, or π-helices as determined by the software DSSP (dictionary of secondary structure of proteins) (36); if the presumably disordered N-terminal 61-residue stretch is excluded, this fraction reaches 90%. The two extracellular segments are suggested to fold into four-helix bundles (with helices H1 to H4 and H1′ to H4′, respectively), which align in parallel to one another and to the membrane normal, intimately interacting with one another. The first and last helices of each bundle are connected to the transmembrane segments of GlnX via linker regions spanning a distance of approximately 15 Å. Intriguingly, for the N-terminal helix of the first bundle and the C-terminal helix of the second, these linkers are all-helical as well, resulting in continuous straight helices spanning almost the entire dimension of the molecule perpendicular to the membrane plane, whereas in the remaining two cases linker helices are kinked and/or noncontiguous. Overall, model confidence of the top-ranked GlnX model was higher for the periplasmic portions than for the other parts of the molecule (Fig. S6A). The pLDDT values are consistently below 50 for N-terminal residues 1 to 72 and tend to increase afterwards, exceeding 75 at residue 90. Consistently, DSSP analysis indicates residues 1 to 61 to be disordered, whereas residues 62 to 86 form a helical segment. Note that the N-terminal residues featuring low pLDDT values also have a poorly defined spatial relationship to the remainder of the molecule, as signified by their high predicted aligned error (PAE) values (Fig. S6B). Consequently, excluding residues 1 to 90 from the prediction results in further improvement of PAE for the well-defined parts of the model (not shown).

## DISCUSSION

In this study, we analyzed the function and properties of the proteins GlnH and GlnX of C. glutamicum, which are encoded together with the gene for the serine/threonine protein kinase PknG in the putative *glnX-glnH-pknG* operon. Deletions of each of these genes individually or of all three genes together led to a strong growth defect when l-glutamine was used as the sole carbon and nitrogen source, and the amount of unphosphorylated OdhI was strongly increased in these strains (Fig. 1). The growth defect can be explained by the inhibition of ODH activity due to binding of unphosphorylated OdhI to the OdhA subunit (6, 8, 9). As a consequence, 2-oxoglutarate generated from glutamine cannot be efficiently oxidized to succinyl-CoA, and carbon flux through the TCA cycle is reduced. Support for this explanation is provided by the finding that the mutants Δ*pknG*, Δ*glnH*, and Δ*glnX* secrete about twice as much l-glutamate as the WT when cultured in the presence of ethambutol, which is known to elicit glutamate overproduction (22). Furthermore, we showed here that the growth defect of the Δ*glnX* mutant can be abolished by the additional deletion of *odhI*. The observation that deletion of the gene cluster *glnX-glnH-pknG* reduced OdhI phosphorylation to a similar extent as the individual deletions of these genes suggests that the three proteins do not have independent, additive effects on the phosphorylation of OdhI but are components of a signal transduction cascade in which GlnH and GlnX regulate the kinase activity of PknG.

Analysis of C. glutamicum Δ*glnX2* suppressor mutants that regained the ability to grow on glutamine agar plates further underlined the role of GlnX in the regulation of OdhI activity. Of 20 analyzed suppressor mutants, 16 were found to carry mutations in the *odhI* gene (Table 1), and in most of them OdhI was no longer detectable by Western blotting. Insertion of stop codons or frameshift mutations led to defective OdhI variants that are likely degraded. Also, amino acid exchanges can cause protein misfolding and degradation, which can explain the reduction or absence of detectable OdhI in the variants E91K, T84I, and F137S (Fig. 3). In these cases, the improved growth of the suppressor mutants on glutamine can be explained by the loss of ODH inhibition. For the suppressor mutant carrying OdhI-R87P, OdhI was still detectable, and our studies with purified OdhI-R87P and OdhI-R87A demonstrated that both variants inhibited ODH activity much less than wild-type OdhI, explaining the restored growth on glutamine (Fig. 4). The structural model of the OdhI-OdhA complex suggested that the R87P and R87A exchanges cause the loss of a predicted salt bridge of R87 with OdhA-D798, probably leading to a lowered binding affinity and thus reduced inhibition of ODH activity. In the case of the suppressor variant OdhI-L107V, reduced hydrophobic interactions with the αE-helix of OdhA might cause a lowered binding affinity and ODH inhibition. These suppressor mutations confirmed the importance of individual residues at the OdhI-OdhA interaction surface for the inhibition of ODH activity.

Use of the cyclic peptide antibiotic globomycin, which specifically inhibits the lipoprotein signal peptidase, and use of a GlnH-C27A mutant protein, in which the attachment of the lipid anchor to C27 is prevented, provided experimental evidence that GlnH is an extracytoplasmic protein attached to the membrane by a typical lipoprotein anchor (Fig. 5). In contrast, the original annotation of GlnH as a glutamine binding protein (20) could not be confirmed, as no binding of l-glutamine to a purified soluble GlnH variant was observed in ITC experiments. Rather, l-aspartate and l-glutamate were shown to bind to GlnH with affinities in the high μM to low mM range (Fig. 6). The results from the ITC experiments are in accordance with findings by Bhattacharyya and coworkers, who reported that l-aspartate and l-glutamate increased the thermal stability of GlnH of C. glutamicum and caused an increase in intrinsic tryptophan fluorescence (21). The *K_D_* values measured by the change in intrinsic tryptophan fluorescence, as reported by these authors (550 ± 90 μM for l-aspartate, 2,060 ± 390 μM for l-glutamate) were somewhat higher than the *K_D_* values measured in our ITC experiments (264 ± 14.6 μM for l-aspartate, 1,256 ± 220 μM for l-glutamate) but showed a similar ratio for l-aspartate and l-glutamate (21). The roughly 2-fold difference in the *K_D_* values are likely caused by differences in the experimental conditions applied.

For M. tuberculosis GlnH, the affinities for l-aspartate and l-glutamate were 50- to 135-fold higher, with *K_D_* values of 4.8 ± 0.6 μM for aspartate and 15.2 ± 5.7 μM for glutamate (21). The strong differences in the ligand binding affinities of the GlnH proteins of these two organisms are surprising and might be explained by the evolutionary adaptation to the different environmental niches of these bacteria. C. glutamicum is an apathogenic species originally isolated from soil, whereas M. tuberculosis is a pathogen able to survive for long time periods inside human phagocytes. Under these conditions, control of the carbon flux at the 2-oxoglutarate node by GarA, the homolog of OdhI in mycobacteria, might require a response to much lower concentrations of l-aspartate and l-glutamate compared to a soil bacterium. From a structural point of view, these differences might be explained by differences in the architecture of the GlnH ligand binding site. A prominent difference is the exchange of Ser and Thr residues crucial for ligand binding. A mutated variant of the C. glutamicum GlnH protein possessing the same arrangement of these residues as that found in the M. tuberculosis protein showed a clear increase in the affinities for l-aspartate and l-glutamate (Fig. 7), demonstrating the importance of the position of these two residues for the ligand binding affinity.

Since the membrane protein GlnX is supposed to act as a transmitter between the extracytoplasmic lipoprotein GlnH and the cytoplasmic kinase PknG, knowledge of the GlnX topology is important to understand its interaction with other proteins. Topology prediction using bioinformatics tools such as Protter (37) and fusion of GlnX variants with commonly used reporters for periplasmic and cytoplasmic localization in bacteria, PhoA and LacZ, provided an experimentally based topology model of GlnX (Fig. 8). According to this model, the large extracytoplasmic segments of GlnX are assumed to be responsible for the proposed interaction with GlnH. Moreover, similarity of the second extracytoplasmic domain with the family of CHASE3 sensor domains found in various classes of transmembrane receptors (32) opens up the possibility that the periplasmic part of GlnX might itself harbor binding sites for small-molecule ligands (38, 39), broadening the sensory capabilities of GlnX beyond the metabolites recognized by GlnH.

The structural model of GlnX created by AlphaFold2 (Fig. 9) not only confirms the conclusions described above but also offers significant new insight into the architecture and functionality of this protein. Schematically, GlnX can be viewed as being composed of three tetra-helical bundles, two constituting the periplasmic portion and the third being inserted into the membrane. The antiparallel arrangement allows for extended helices to be stable despite their significant dipole moment. Indeed, the longest contiguous helix (67 amino acid [aa] residues), comprising the first transmembrane segment, the ensuing linker, and helix H1 of the first bundle, extends over approximately 100 Å. A sequence-based search for related structures in the Protein Data Bank (PDB) using profile hidden Markov models (HMMER [40]) did not yield obvious hits. In contrast, structural comparison via Dali (41) revealed that the periplasmic portion actually represents a well-established fold. Its two four-helix bundles closely resemble the 4HB sensory module originally described for the aspartate receptor Tar of Salmonella enterica serotype Typhimurium (42) and later found in a variety of other chemoreceptors and sensory histidine kinases. Indeed, for both periplasmic domains of GlnX, the ligand binding domain of the citrate-sensing chemoreceptor MCP2201 from Comamonas testosteroni (PDB code 6ITS) and the periplasmic domain of the transmembrane histidine kinase LytS from Clostridium beijerinckii (PDB code 5XSJ) are among the most significant structural matches. In the Pfam database, the 4HB fold is represented by the 4HB_MCP superfamily, comprising four distinct families (4HB_MCP_1, CHASE3, TarH, and HBM). While the second periplasmic domain of GlnX matches CHASE3, albeit with borderline significance (E value, 9.5 × 10^−5^), no match was found for the first periplasmic domain, indicating that its sequence has further diverged from the presumed common 4HB ancestor while the characteristic three-dimensional fold was preserved. Indeed, despite insignificant sequence similarity, the two bundles superimpose very well; secondary structure matching (SSM [43]) using segments 128–255 and 330–460 yields an RMSD of 1.9 Å for 117 aligned Cα atoms. Note that the second periplasmic domain is distinguished by an eight-residue insertion into helix H4′, forming a finger-like protrusion with three acidic residues (^435^DEE^437^) at its tip (Fig. 9). This portion of GlnX seems poised for interaction with other proteins, an obvious candidate being GlnH (see below).

Periplasmic 4HB domains flanked by transmembrane segments usually occur as a single copy per chain in prokaryotic chemoreceptors and histidine kinases but display a strong tendency to dimerize. These dimers are already found in the apo state but are often stabilized by ligand binding at the interface of the two domains. The helical bimodular (HBM) family represents an exception in that it features two helical domains per chain, with the second being inserted into the first, such that only one retains its connection to membrane-spanning helices. Consequently, receptor dimerization leads to two 4HB dimers in a stacked arrangement. While GlnX is also predicted to contain two periplasmic helical domains, its architecture fundamentally differs from the HBM type. GlnX appears to have evolved by duplication of a canonical 4HB domain, including its flanking membrane anchors; the resulting arrangement closely resembles the noncovalent dimer formed by Tar and similar chemoreceptors, as far as the periplasmic and transmembrane portions are concerned (Fig. S7). Indeed, the two periplasmic helical domains in our GlnX model are related by a rotation of 171°, which is well within the range found for 4HB homodimers in crystal structures if protomers are related by noncrystallographic symmetry. The interaction involves more than 1,000 Å^2^ of surface area on either domain and is stabilized by numerous polar and apolar contacts. To our knowledge, this tandem arrangement of membrane-anchored 4HB domains on a single chain has not been reported for any other protein. In order to distinguish it from the four known 4HB families in Pfam, with which GlnX shares at most borderline sequence similarity (see above), we suggest naming this unique architecture a helical tandem module (HTM).

The actual mechanism by which GlnX receives its input signals and transduces them across the membrane is still under investigation. Importantly, the involvement of an additional protein in the sensing process is not unprecedented in the 4HB family; Tar, e.g., is known to interact with maltose binding protein (MBP), thereby acquiring sensitivity to maltose in addition to aspartic acid, which interacts directly with the 4HB domains. Indeed, both GlnH and MBP belong to the periplasmic binding protein (PBP) superfamily (Pfam families SBP_bac_3 and SBP_bac_1, respectively) and share the same tertiary structure. A potential mode of interaction is indicated by the X-ray structure of the LytS periplasmic domain complexed with its cognate d-xylose sensor XylFII (44). Here, the ligand binding XylFII protein associates with helices H3 and H4 of the LytS 4HB module, with the long axes of both molecules arranged approximately at a right angle. While XylFII and GlnH are at most distantly related, we note that superposition of the LytS-XylFII complex onto our GlnX model positions the periplasmic binding protein in the immediate vicinity of the acidic H4′ finger of GlnX (Fig. S8).

The mode of signal propagation through the membrane used by 4HB sensors is complex and still subject to active research; current evidence points to ligand binding causing (among other changes) a piston-like displacement of helix H4 in the periplasmic sensor module, which may either propagate as such into the transmembrane helix bundle or transform into different modes, such as helical rotation or scissoring (45). Given the architectural similarities of GlnX with the periplasmic and transmembrane portions of a classical dimeric chemoreceptor, it seems reasonable to assume that similar principles apply. On the other hand, the cytoplasmic part of GlnX does not contain any of the domains typically found in bacterial sensors (such as histidine kinase and receiver domains, HAMP domains [present in histidine kinases, adenylyl cyclases, methyl accepting proteins and phosphatases], and kinase control modules), and its mode of interaction with its effector PknG is still elusive.

**FIG 10:**
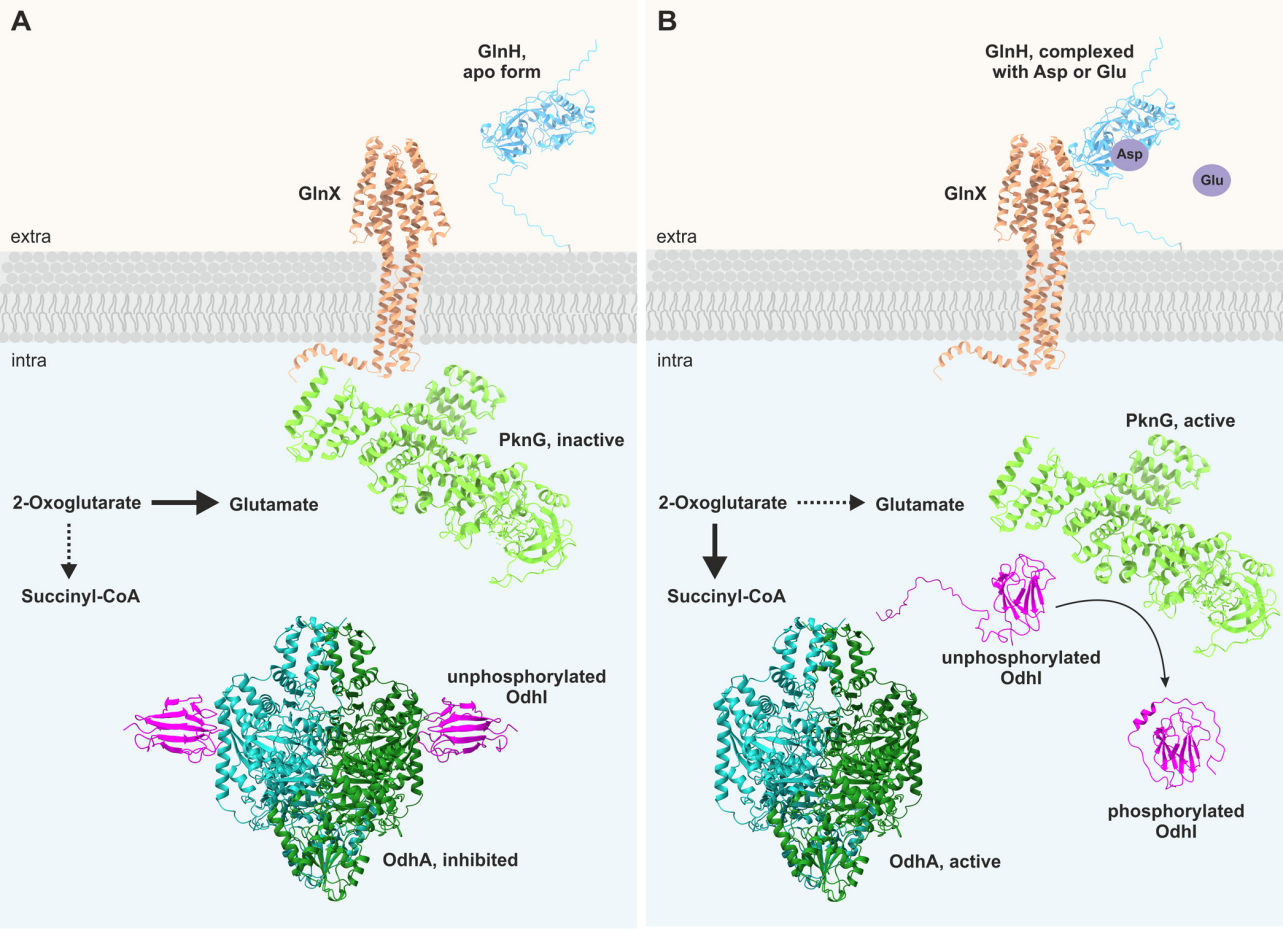
Hypothetical model of the GlnH-GlnX-PknG-OdhI-OdhA signal transduction cascade. Note that the assumed interactions of GlnH with GlnX and of GlnX with PknG have not been demonstrated yet. (A) In the absence of l-aspartate or l-glutamate in the periplasm, PknG forms a complex with the cytoplasmic parts of GlnX and is inactive. OdhI is predominantly unphosphorylated, binds to the OdhA subunit of ODH, and inhibits its activity, shifting the flux of 2-oxoglutarate toward l-glutamate and ammonium assimilation. (B) In the presence of l-aspartate or l-glutamate in the periplasm, GlnH binds these amino acids and interacts with the periplasmic tandem four-helix bundle domain of GlnX. In addition, this domain of GlnX might itself also serve as a receptor to bind yet unknown ligands. These binding events are signaled across the membrane via contiguous α-helices (H1-TMH1 and/or H4′-TMH4) and trigger a conformational change of PknG that causes activation of the kinase activity and dissociation from GlnX. Active PknG phosphorylates OdhI at Thr14, triggering a conformational change of OdhI that abolishes its interaction with OdhA. Consequently, the inhibition of ODH activity is relieved and the flux of 2-oxoglutarate is shifted toward the TCA cycle and energy generation. The structures for GlnH, GlnX, and the OdhI-OdhA complex represent AlphaFold2 models generated in this work. The structure of PknG (PDB code 7MXB [13]) and the structures of unphosphorylated and phosphorylated OdhI (PDB codes 2KB4 and 2KB3, respectively [11]) were determined experimentally.

Taken together, all these findings support a signal transduction model in which the phosphorylation status of OdhI is controlled by a signal transduction cascade consisting of GlnH, GlnX, and PknG (Fig. 10). In this model, the presence of l-glutamate and l-aspartate in the periplasm is sensed by GlnH. GlnH is assumed to interact with the periplasmic domains of GlnX, with the acidic protrusion in helix H4′ being an attractive interaction site. In its ligand-bound state, GlnH is proposed to trigger a conformational change of GlnX, enabling the transfer of the information on the ligandation status of GlnH across the cytoplasmic membrane. The cytoplasmic parts of GlnX are proposed to interact with the tetratricopeptide repeat domain of PknG (13, 21), triggering a conformational change of PknG that activates the kinase activity and causes the dissociation of PknG from GlnX. The phosphorylation of threonine residues in the N-terminal region, which stabilize the interaction with the FHA substrate (13), may play a role in this process. Such a model could explain the observation that PknG was detected both in the membrane fraction and in the cytosolic fraction of M. tuberculosis (15) and C. glutamicum (our unpublished data). Active PknG then phosphorylates OdhI on T14, thereby abolishing the inhibition of ODH activity by unphosphorylated OdhI. As a consequence, the carbon flux from 2-oxoglutarate is shifted from glutamate synthesis toward the TCA cycle. l-glutamate is the major amino group donor for amino acids in cells and the metabolite with the by far highest concentration (~100 mM) in the cytoplasm of C. glutamicum (46). It can be taken up by the ABC transporter GluABCD (47) or the Na^+^-coupled secondary transporter GltS (48), thereby reducing the requirement for glutamate synthesis and providing more energy by 2-oxoglutarate catabolism in the TCA cycle. Control of carbon flux at the 2-oxoglutarate node by external glutamate thus appears reasonable. For l-aspartate, no specific uptake system has been described yet in C. glutamicum, and its role as nitrogen donor is restricted compared with l-glutamate. Therefore, the reason why l-aspartate may control the OdhI phosphorylation status is not as obvious as for l-glutamate. In summary, the GlnH-GlnX-PknG-OdhI-OdhA signal transduction cascade can be considered a component of the regulatory network controlling the balance between nitrogen assimilation and carbon metabolism in *Corynebacterium* and probably many other members of the *Actinobacteria*, such as Mycobacterium species.

## MATERIALS AND METHODS

### Bacterial strains, media, and culture conditions.

All bacterial strains and plasmids used in this work are listed in Table 2. Escherichia coli cells were cultivated at 37°C in lysogeny broth (LB) (49) or terrific broth (TB) (12 g L^−1^ tryptone, 24 g L^−1^ yeast extract, 4 mL glycerol, 12.54 g L^−1^ K_2_HPO_4_, 2.31 g L^−1^ KH_2_PO_4_; pH 7.0) or on LB agar plates (Carl Roth, Karlsruhe, Germany). C. glutamicum strains were cultivated at 30°C in brain heart infusion medium (BHI; Difco Laboratories, Detroit, USA), in CGXII medium with 4% (wt/vol) glucose (50) containing 30 mg L^−1^ 3,4-dihydroxybenzoate as the iron chelator, or in modified CGXII medium lacking glucose, ammonium sulfate, and urea and containing 100 mM l-glutamine as the nitrogen and carbon source. These media were also used for the preparation of solid media by addition of 15 g L^−1^ agar. For induction of glutamate secretion, the CGXII medium with 4% (wt/vol) glucose was supplemented with 500 mg L^−1^ ethambutol and 20 μM IPTG for strains carrying plasmids with an IPTG-inducible promoter. The glutamate concentration in the culture supernatant was measured after 24 h. To maintain plasmid stability, kanamycin was added at concentrations of 25 mg L^−1^ (C. glutamicum) or 50 mg L^−1^ (E. coli).

**TABLE 2:** Bacterial strains and plasmids used in this study

Strain or plasmid	Description	Reference or source
C. glutamicum strains		
WT	ATCC13032; biotin-auxotrophic WT strain	DSMZ
Δ*pknG*	WT derivative with an in-frame deletion of *pknG* (cg3046)	6
Δ*glnH*	WT derivative with an in-frame deletion of *glnH* (cg3045)	6
Δ*glnX2*	WT derivative with an in-frame deletion of *glnX* (cg3044); 59 codons at the 5′ end and 142 codons at the 3′ end were kept; the 301 codons in between were deleted and replaced by an artificial 21-bp sequence	This work
Δ*glnX-glnH-pknG*	WT derivative with in-frame deletion of *glnX-glnH-pknG* (cg3044-cg3046)	This work
Δ*odhI*	WT derivative with in-frame deletion of *odhI* (cg1630)	6
Δ*glnX2* Δ*odhI*	WT derivative with in-frame deletions of *odhI* (cg1630) and *glnX* (cg3044)	This work
E. coli strains		
DH5α	F- *supE44* Δ*lacU*169 (Φ80*lacZ*Δ*M15*) *hsdR17 recA1 endA1 gyrA96 thi-1 relA1*	53
BL21(DE3)	F- *ompT hsdSB(rB-mB-) gal dcm* (λcIts857) *ind1* Sam7 *nin5 lacUV5*-T7 Gen 1	67
TG1	F- *supE thi*-1 Δ(*lac-proAB*) Δ(*mcrB-hsdSM*)5(rK-mK-)(*traD36 proAB* + *lacI*q *lacZΔM15*)	Lucigen Corporation
Plasmids		
pK19mobsacB	Kan^R^; suicide vector for allelic exchange in C. glutamicum; *oriV*_Ec_ *oriT sacB*	68
pK19mobsacB-*glnX*2	Kan^R^; pK19mobsacB derivative containing PCR products (primer ΔglnX_1–4) covering the up- and downstream regions of the *glnX* gene	This work
pK19mobsacB-*glnX*-*glnH*-*pknG*	Kan^R^; pK19mobsacB derivative containing PCR products (primer ΔglnX 1+2 and ΔpknG 3+4) covering the up- and downstream regions of the *glnX and pknG* genes, respectively	This work
pK19mobsacB-*odhI*	Kan^R^; pK19mobsacB derivative containing PCR products covering the up- and downstream regions of the *odhI* gene	6
pET-TEV	Kan^R^; pET28b derivative for protein overproduction in E. coli, contains a His_10_-tag and a TEV cleavage site	69
pET-TEV-*odhI*	Kan^R^; pET-TEV derivative for overproduction of OdhI with an N-terminal His_10_-tag and TEV cleavage site	8
pET-TEV-*odhI*-R87A	Kan^R^; pET-TEV-*odhI* derivative, encodes OdhI-R87A with an N-terminal His_10_-tag and TEV cleavage site	This work
pET-TEV-*odhI*-R87P	Kan^R^; pET-TEV-*odhI* derivative, encodes OdhI-R87P with an N-terminal His_10_-tag and TEV cleavage site	This work
pET-TEV-*glnH*ΔSP	Kan^R^; pET-TEV derivative for overproduction of GlnH lacking the signal peptide and the lipobox motif with an N-terminal His_10_-tag and TEV cleavage site	This work
pET-TEV-*glnH*core	Kan^R^; pET-TEV derivative for overproduction of GlnH lacking flexible N- and C-terminal parts (amino acid residues 48–334 of GlnH) with an N-terminal His_10_-tag and TEV cleavage site	This work
pET-TEV-*glnH*core-S163T-T165S	Kan^R^; pET-TEV-*glnH*core derivative, encodes GlnHcore-S163T-T165S with an N-terminal His_10_-tag and TEV cleavage site	This work
pJC1	Kan^R^; E. coli-C. glutamicum shuttle vector	70
pJC1-*glnX*Prom	Kan^R^; pJC1 derivative carrying the *glnX* gene, including its native promoter region (383 bp upstream of the transcriptional start site, which is also the translational start site)	This work
pEKEx2	Kan^R^, E. coli/C. glutamicum shuttle vector; P_*tac*_; *lacI*^q^; *ori_Cg_* from pBL1; *ori_Ec_* ColE1 from pUC18	71
pEKEx2-*pknG*	Kan^R^, pEKEx2 derivative encodes PknG with a C-terminal Strep-tag, contains the native RBS^*a*^ of *pknG*	6
pAN6	Kan^R^; C. glutamicum/E. coli shuttle vector for regulated gene expression	72
pAN6-*glnH*	Kan^R^; pAN6 derivative encodes GlnH with a C-terminal Strep-tag	This work
pAN6-*glnH*-C27A	Kan^R^; pAN6-glnH derivative, encodes GlnH-C27A with a C-terminal Strep	This work
pPREx2	Kan^R^; pPBEx2 derivative (P*_tac_*, *lacI*^q^, *oriC.g* from pBL1; *ori_Ec_* ColE1 from pUC18), with a consensus RBS (AAGGAG) for C. glutamicum	35
pPREx2-*glnX*-(F1 to F7)-*phoA*	Kan^R^; pPREx2 derivative for expression of GlnX variants of different lengths (F1–F7) fused to the alkaline phosphatase (*phoA*)	This work
pPREx2-*glnX*-(F1 to F7)-*lacZ*	Kan^R^; pPREx2 derivative for expression of GlnX variants of different lengths (F1–F7) fused to the β-galactosidase (*lacZ*)	This work
pMA632-Ex	Amp^R^; E. coli plasmid for topology determination of membrane proteins via *phoA*-*lacZ* fusions *(P_tac_, lacI^q^*; with RBS) used as template for *phoA* amplification including linker sequence	Provided by Lothar Eggeling, Forschungszentrum Jülich

a RBS, ribosomal binding site.

### Standard recombinant DNA work and construction of deletion mutants.

Standard methods such as PCR and plasmid restriction were carried out according to established protocols (51). All oligonucleotides used are listed in Table 3. Plasmids were constructed by ligating DNA fragments obtained by restriction digestion or PCR or by Gibson assembly (52). Oligonucleotide synthesis and DNA sequencing were performed by Eurofins Genomics (Ebersberg, Germany). Site-directed mutagenesis of *odhI* and *glnH* was performed using the QuikChange Lightning site-directed mutagenesis kit (Agilent Technologies, Santa Clara, CA, USA). Transformation of E. coli was performed using a standard protocol (53), and C. glutamicum transformation was performed by electroporation (54). C. glutamicum deletion mutants were constructed by double homologous recombination using pK19mobsacB-based plasmids as described previously (55). Oligonucleotides annealing up- and downstream of the deleted genes were used to confirm genomic deletions by colony-PCR.

**TABLE 3:** Oligonucleotides used in this study

Name	Sequence
Construction of pK19mobsacB-*glnX2* and pK19mobsacB-*glnX-glnH-pknG*	
ΔglnX_1	TATATAGTCGACGCGTTCTGGATCGGCGGGAAAAGG
ΔglnX_2	CCCATCCACTAAACTTAAACAAGAAGTATCCTTCTCCGTTAACGG
ΔglnX_3	TGTTTAAGTTTAGTGGATGGGTCGATTCAGGGCAGTGGCACTGGT
ΔglnX_4	TATATAGGATCCAAAGCAGTCAAGGTTTCTGCCGGT
ΔpknG_3	TGTTTAAGTTTAGTGGATGGGGCGAATGCCGTGCGG
ΔpknG_4	GATTCTAGAGTTAGCTATCGCTAGGTACG
Confirmation of gene deletion by colony-PCR	
ΔglnX_fw	ATTCATCAAGGTAGCCAATACTTTC
ΔglnX_rv	GCTCCTCAGGGGTCAGATCAT
ΔpknG_rv	GATCGATCCAGAGCGTAACGC
Construction of pJC1-*glnX*Prom	
glnXProm-fw	TATAGGATCCGCGTTCTGGATCGGCGGGAAAAG
glnXProm-rev	TATAGTCGACTTATAAGTACTCCTGCAAACGGGGG
Construction of pET-TEV-*odhI*-R87A and pET-TEV-*odhI*-R87P by site-directed mutagenesis	
odhI_R87A fw	CTTGATGATGTCACCGTTTCAGCTCGCCACGCAG
odhI_R87A rv	CTGCGTGGCGAGCTGAAACGGTGACATCATCAAG
odhI_R87P fw	TGTCACCGTTTCACCTCGCCACGCAGAG
odhI_R87P rv	ACAGTGGCAAAGTGGAGCGGTGCGTCTC
Construction of pET-TEV-*glnH*ΔSP	
glnHΔSP fw	TATACATATGACTCCAACACCTGTGGAACCG
glnHΔSP rv	TATACTCGAGTTATCCTTCATCGTTTTCTGT
Construction of pET-TEV-*glnH*core	
glnHcore fw	CCTGTATTTTCAGGGCCATATGCCACTGCCACCGGATTCTTC
glnHcore rv	TGGTGGTGGTGGTGGTGCTCGAGTTATGGCATGTACTGCAGCTGTG
Construction of pET-TEV-*glnH*core-S163T-T165S	
glnH_S163T-T165S fw	ATGTAGATATTGTGATTCGTACGGTCTCCATCACCGACGAACGCGCC
glnH_S163T-T165S rv	GGCGCGTTCGTCGGTGATGGAGACCGTACGAATCACAATATCTACAT
Construction of pAN6-*glnH*	
glnH fw	GACAGTCATATGCACGCTTTTCGACGC
glnH rv	GACGCTAGCTCCTTCATCGTTTTCTGTC
Construction of pAN6-*glnH*-C27A by site-directed mutagenesis	
glnH_C27A fw	AACGCTGCTTGCTTCCGCCACTCCAACACCTGTG
glnH_C27A rv	CACAGGTGTTGGAGTGGCGGAAGCAAGCAGCGTT
Construction of pPREx2-*glnX*-(F1 to F7)-*phoA* and pPREx2-*glnX*-(F1 to F7)-*lacZ*	
glnX fw	TGCAGAAGGAGATATACATATGATCCGGGATGGAAATG
glnX-F1 rv	CGTCTGGCCTCTCGAGGCAGGCTGGCCGAAAGTTTCCTG
glnX-F2 rv	CGTCTGGCCTCTCGAGGCAACAGGCTCCGCATTAGTG
glnX-F3 rv	CGTCTGGCCTCTCGAGGCGTTGTAAAGCTCAGACGCCATC
glnX-F4 rv	CGTCTGGCCTCTCGAGGCCCTGCGCGTAATCCGCATCAAC
glnX-F5 rv	CGTCTGGCCTCTCGAGGCATTCAACGGCCCCGACGCTTC
glnX-F6 rv	CGTCTGGCCTCTCGAGGCGCGAGAATCCGCGATCAG
glnX-F7 rv	CGTCTGGCCTCTCGAGGCTAAGTACTCCTGCAAACG
lacZ fw	GCCTCGAGAGGCCAGACGGGCCAGGCCTTGGGCCCTACCATGATTACGGATTCAC
lacZ rv	GTAAAACGACGGCCAGTGTTATTTTTGACACCAGACCAAC
phoA fw	GCCTCGAGAGGCCAGACG
phoA rv	GTAAAACGACGGCCAGTGTTATTTCAGCCCCAGGGCGGCTTTC	
Amplification and sequencing primer for *odhI*, *odhA*, and *pknG*
odhI-78b-up	CAGGAAATTCTAGGATCTTACGGA
odhI-76b-dwn	GGCATTCTATACACAAAACGGTTG
odhA-up80b-fw	AGCCAACGACCAACGTTACAG
odhA-988b-rv	TGCGCAGGAATTCACCGGA
odhA-862b-fw	TGTCGGTTCCATGGATTACC
odhA-2051b-rv	GTGGTGAAGCCGATCTGGTT
odhA-1982-fw	CCAGAAACCATCAACCTGGC
odhA-dwn75b-rv	GCAGCAAAAAGGGCCGTATGCTGTGT
pknG-up150b-fw	GGTTGATTCGGCAGGTAAACTAC
pknG-1300b-rv	GTTTGCCGTCGCGGACTGCC
pknG-1052b-fw	CGATATTTTCACCATCGGACGC
pknG-dwn150b-rv	CGCAGAAATCACATCAGCCCCAAT

### Western blot analysis.

The phosphorylation status of OdhI in different C. glutamicum strains was analyzed by Western blotting. Cell pellets of C. glutamicum strains cultivated overnight in BHI medium with 4% (wt/vol) glucose were resuspended in phosphate-buffered saline (PBS) (137 mM NaCl, 2.7 mM KCl, 4.3 mM Na_2_HPO_4_, 1.4 mM KH_2_PO_4_) containing cOmplete EDTA-free protease inhibitor (Roche, Basel, Switzerland) and disrupted using 0.1-mm zirconia/silica beads (Roth, Karlsruhe, Germany) in an amalgamator (Silamat S5, Vivadent). Cell lysates were cleared by centrifugation at 13,000 × *g* and 4°C for 45 min followed by ultracentrifugation at 100,000 × *g* and 4°C for 1 h. Samples of the supernatants corresponding to 20 μg protein were analyzed using SDS-PAGE and Western blotting as previously described (14). The presence of OdhI in the C. glutamicum Δ*glnX2* strain and its suppressor mutants was analyzed with crude cell extract samples (10 μg protein each) using polyclonal rabbit anti-OdhI antiserum in a 1:500 dilution (14). The ECL Advance Western blotting detection kit (GE Healthcare, Chicago, IL, USA) was used for chemiluminescent signal detection using an LAS-3000 image reader (Fujifilm, Minato, Japan). Signal intensity was analyzed using ImageQuant software (GE Healthcare) or AIDA Image Analyzer v. 4.15 (Fujifilm).

To analyze if GlnH is a lipoprotein, the C. glutamicum strains Δ*glnH*/pAN6-*glnH* and Δ*glnH*/pAN6-*glnH*-C27A were cultivated in CGXII medium with 4% (wt/vol) glucose and 1 mM IPTG to induce target gene expression. After 4 h of cultivation (optical density at 600 nm [OD_600_], ~6), 50 mg L^−1^ globomycin (stock solution, 20 g L^−1^ in chloroform) was added, and the culture was further incubated for 3 h at 30°C. In the control culture, no globomycin was added. Crude cell extract samples (10 μg protein) taken prior to addition of globomycin and 1 h, 2 h, and 3 h thereafter were analyzed by Western blotting using a Strep-Tactin-horseradish peroxidase (HRP) conjugate (IBA Lifesciences, Göttingen, Germany) to detect Strep-tagged GlnH.

### Determination of glutamate.

The concentration of l-glutamate in culture supernatants was determined by reversed-phase high-pressure liquid chromatography as reported previously for l-lysine (56).

### Protein production and purification.

For measuring their influence on ODH activity, His-tagged OdhI and the mutated derivatives OdhI-R87P and OdhI-R87A were overproduced using the E. coli strains BL21(DE3)/pET-TEV-*odhI*, BL21(DE3)/pET-TEV-*odhI*-R87P, and BL21(DE3)/pET-TEV-*odhI*-R87A. The strains were cultivated at 37°C in LB medium, and at an OD_600_ of 0.5, target gene expression was induced with 1 mM IPTG, after which the cultures were incubated for another 4 h at room temperature. His_10_-GlnHΔSP, His_10_-GlnHcore, and its mutated derivative His_10_-GlnHcore-S163T-T165S were overproduced in TB medium using E. coli BL21(DE3) containing the plasmids pET-TEV-*glnH*ΔSP, pET-TEV-*glnH*core, and pET-TEV-*glnH*core S163T-T165S. After target gene expression with 500 μM IPTG, the cells were cultivated for 18 h at 18°C. All proteins were purified following the same protocol. Cells were harvested by centrifugation, resuspended in lysis buffer (20 mM Tris-HCl, 500 mM NaCl, 5% [vol/vol] glycerol, 20 mM imidazole, pH 7.9) containing cOmplete EDTA-free protease inhibitor (Roche, Basel, Switzerland), and disrupted by French press treatment. Soluble protein fractions were obtained by centrifugation (5,000 × *g*, 4°C, 20 min) and subsequent ultracentrifugation of the supernatant (100,000 × *g*, 4°C, 1 h). Supernatants of the ultracentrifugation were loaded on HisTrap HP (GE Healthcare, Chicago, IL, USA) or gravity flow Ni-NTA columns (Qiagen, Hilden, Germany), and after washing, the His-tagged proteins were eluted using buffer with increasing imidazole concentrations up to 300 mM. In the case of purified OdhI variants, His-tags were cleaved off by addition of His-tagged *Tobacco Etch Virus* (TEV) protease (0.01 mg/mg target protein) and overnight incubation at 4°C followed by a second Ni-NTA purification, in which untagged OdhI was collected in the flowthrough. Untagged OdhI variants and His-tagged GlnH variants were further purified by size exclusion chromatography on a Superdex 200 10/300 GL column (GE Healthcare) equilibrated for the OdhI variants in 0.1 M TES [*N*-tris(hydroxymethyl)methyl-2-aminoethanesulfonic acid] buffer (100 mM TES, pH 7.2, 10 mM MgCl_2_, 3 mM cysteine, 30% [vol/vol] glycerol), and for GlnH variants in HEPES buffer (40 mM HEPES, pH 7, 100 mM KCl, 10 mM MgCl_2_). Protein concentrations were determined using the molar extinction coefficient predicted by the ProtParam tool (http://web.expasy.org/protparam/).

### Enzyme activity assays.

Determination of the ODH activity in the presence of different OdhI variants was carried out using a photometric assay following NADH formation as described previously (6, 8). Native OdhI, OdhI-R87A, or OdhI-R87P at concentrations of 0.38 nM or 1.9 nM were added to cell-free extracts of C. glutamicum Δ*odhI* and preincubated for 5 min at room temperature and then for 3 min at 30°C in the spectrophotometer before the reaction was started by addition of 0.2 mM coenzyme A.

For the determination of alkaline phosphatase (PhoA) and β-galactosidase (LacZ) activity of GlnX′-PhoA and GlnX′-LacZ fusion constructs, E. coli TG1 cells were transformed with pPREx2 plasmids encoding seven GlnX variants of different lengths fused either to PhoA or to LacZ. The strains (in biological triplicates) were cultivated in LB medium in 96-well deep-well plates at 30°C and 900 rpm for 3 h, and expression of the *glnX-phoA* and *glnX-lacZ* constructs were induced by addition of 1 mM IPTG. After further incubation for 1 h under the same conditions, 100 μL of each culture was harvested. Cells carrying the GlnX-PhoA constructs were resuspended in 300 μL 1 M Tris-HCl, pH 8, while those harboring the GlnX-LacZ constructs were resuspended in 300 μL buffer Z (60 mM Na_2_HPO_4_, 40 mM NaH_2_PO_4_, 1 mM MgSO_4_, 10 mM KCl, 100 mM dithiothreitol [DTT], pH 7) (57). Resuspended cells were permeabilized, and activity assays were carried out as previously described (34, 58). PhoA activity was measured with *p*-nitrophenylphosphate (*p*NPP) (5 g L^−1^ *p*NPP in 1 M Tris-HCl, 5 mM MgCl_2_, pH 8), while LacZ activity was assayed with *o*-nitrophenol-β-d-galactopyranoside (*o*NPG) (5 g L^−1^ *o*NPG in 100 mM K_2_HPO_4_, pH 7). The activities were measured by following the changes in the absorbance at 420 nm for up to 2 h at 28°C using a Tecan microplate reader. Prior to the measurement at 420 nm, the absorbance at 550 nm and 600 nm was measured to correct for differences in cell density and light scattering caused by cell debris. The activity in Miller units was calculated according to the equation MU = 1,000 × ([*A*_420_ – 1.75 × *A*_550_]/[*t* × *A*_600_]), where *A*_420_ represents the absorbance of the yellow nitrophenolate, *A*_550_ corrects for light scattering from cell debris, *A*_600_ represents the cell density, and *t* is the reaction time.

### Isothermal titration calorimetry.

Purified His_10_-GlnHΔSP was dialyzed overnight against a 500-fold excess of dialysis buffer (40 mM HEPES, 100 mM KCl, 10 mM MgCl_2_, pH 7.0). Stock solutions of the potential ligands l-aspartic acid, l-glutamic acid, l-asparagine, l-glutamine, and 2-oxoglutarate were prepared in dialysis buffer, and the pH was adjusted to pH 7.0 using KOH. ITC measurements were performed with a MicroCal PEAQ-ITC instrument (Malvern Panalytical, Malvern, Great Britain) operated at 25°C. Protein concentrations between 30 μM and 75 μM were used together with ligand concentrations of 7.5 mM to 50 mM. Prior to being filled with 300 μL protein solution, the measuring cell was rinsed with dialysis buffer, while the syringe was filled with 75 μL ligand solution. An ITC run was started with an initial injection of 0.4 μL followed by 12 injections of 3 μL each. In addition, control experiments with ligand solution titrated into the dialysis buffer were performed. The data were analyzed using MicroCal ITC analysis software (Malvern Panalytical), and a fixed 1:1 binding stoichiometry was assumed to determine the binding affinity.

### Measurement of the effects of amino acids on intrinsic tryptophan fluorescence of GlnH.

Intrinsic fluorescence (excitation at 292 nm, emission at 340 nm) of His_10_-GlnHcore and His_10_-GlnHcore-S163T-T165S (0.1 mg/mL) was measured in 40 mM HEPES buffer, pH 7.0, containing 100 mM KCl and 10 mM MgCl_2_ at various concentrations of sodium aspartate (0.0122 to 50 mM) or sodium glutamate (0.0122 to 100 mM). Fluorescence was measured using 15-μL samples in a Tecan Infinite M1000 PRO plate reader (Tecan, Männedorf, Switzerland) and 384 black shallow-well plates. The dissociation constant of GlnH for amino acids was calculated by fitting fluorescence data to a one-site specific binding equation (GraphPad Prism 8).

### Prediction of GlnH and GlnX structures.

The recent developments in the field of protein structure prediction, most notably the deep learning systems AlphaFold2 (30, 59) and RoseTTAFold (60), have greatly enhanced the accuracy of the resulting models. In the case of AlphaFold2, structural models of proteins not yet represented in the respective database (https://alphafold.ebi.ac.uk) became more approachable through the availability of the open-source software ColabFold (61), featuring improved speed and resource efficiency. Furthermore, AlphaFold2 has also been reported to be reliable for predicting transmembrane protein structures (62). The monomeric structures of GlnH and GlnX were predicted by AlphaFold2 (30) via the ColabFold interface (30, 59, 63), using the GlnHΔSP sequence (residues 28 to 343) and the full-length GlnX sequence of 501 amino acids, respectively. The pipeline executed for prediction in ColabFold generates a multiple-sequence alignment (MSA) via an MMseqs2 search (64); the MSA was subsequently used as input for the prediction [parameters use_amber: no, template mode: none, msa_mode: MMSeq2 (UniRef+Environmental], num_recycle: 12). The structure was visualized using ChimeraX (65) or PyMol (www.pymol.org) and colored, when appropriate, according to the pLDDT confidence measure annotated in the B-factor field of the PDB file.
