# Supplementary material for: Characteristics of the GlnH and GlnX Signal Transduction Proteins Controlling PknG-Mediated Phosphorylation of OdhI and 2-Oxoglutarate Dehydrogenase Activity in Corynebacterium glutamicum
Source: Microbiol Spectr. 2022 Nov 29;10(6):e02677-22. doi: 10.1128/spectrum.02677-22 (PMC9769921; doi:10.1128/spectrum.02677-22)
Supplement: Supplemental file 1 — Supplemental material. Download spectrum.02677-22-s0001.pdf, PDF file, 0.9 MB [file spectrum.02677-22-s0001.pdf]

Supplementary information

Characteristics of the GlnH and GlnX signal transduction proteins controlling PknG-mediated phosphorylation of OdhI and 2-oxoglutarate dehydrogenase activity in *Corynebacterium glutamicum*

Lea Sundermeyer[1#], Graziella Bosco[1#], Srushti Gujar[1,2,3], Melanie Brocker[1], Meike Baumgart[1], Dieter Willbold[2,3], Oliver H. Weiergräber[2], Marco Bellinzoni[4], and Michael Bott[1,5]*

[#]Lea Sundermeyer and Graziella Bosco contributed equally to this work.

[1]IBG-1: Biotechnology, Institute of Bio- and Geosciences, Forschungszentrum Jülich, 52425 Jülich, Germany

[2]IBI-7: Structural Biochemistry, Institute of Biological Information Processing, Forschungszentrum Jülich, 52425 Jülich, Germany

[3]Institut für Physikalische Biologie, Heinrich-Heine-Universität Düsseldorf, 40225 Düsseldorf, Germany

[4]Unité de Microbiologie Structurale, Institut Pasteur, CNRS UMR 3528, Université de Paris, Paris, France

[5]Bioeconomy Science Center (BioSC), Forschungszentrum Jülich, 52425 Jülich, Germany

*Corresponding author: email m.bott@fz-juelich.de; phone +49 2461 613294

Running title: GlnH-GlnX-PknG-OdhI-OdhA signal transduction cascade

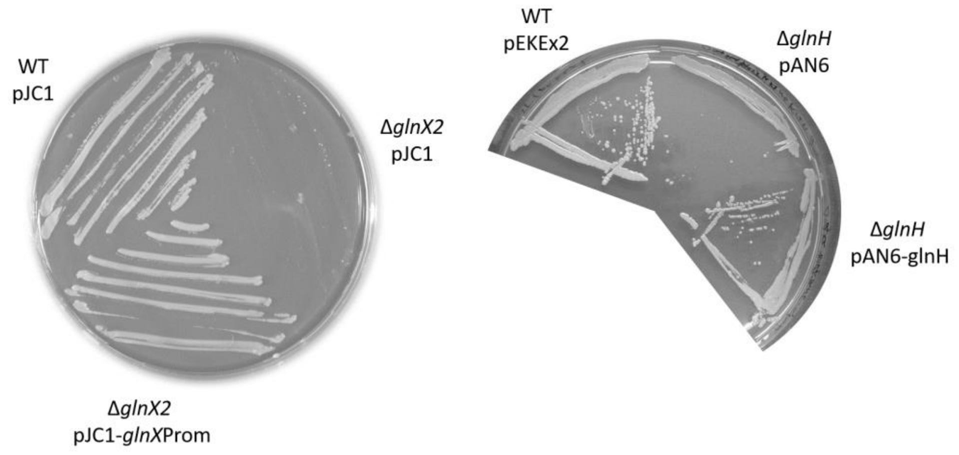

Fig. S1. Complementation of the growth defect on glutamine agar plates of the *C. glutamicum* mutants Δ*glnX2* and Δ*glnH* by plasmid-based expression of *glnX* or *glnH*, respectively.

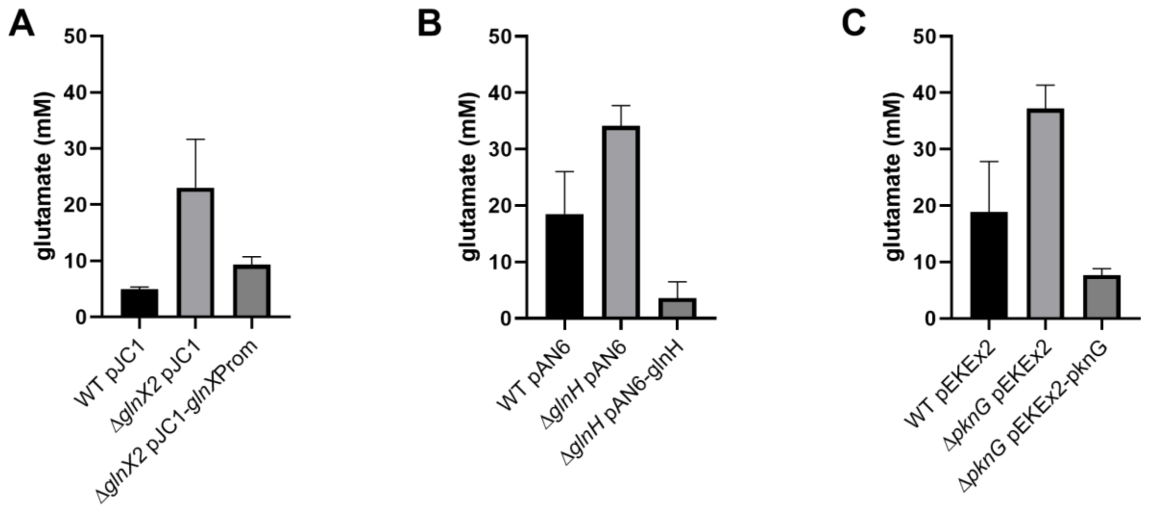

Fig. S2. Glutamate excretion triggered by ethambutol in *C. glutamicum* WT and the mutant strains Δ*pknG*, Δ*glnH*, and Δ*glnX2*. The strains were cultivated in CGXII medium with 4% (w/v) glucose supplemented with 500 mg/l ethambutol and 25 mg/l kanamycin. In experiments B and C, the medium contained 20 µM IPTG in addition. The glutamate concentration in the culture supernatants was measured after 24 h of cultivation. Shown are average values and standard deviations of at least three independent cultivations.

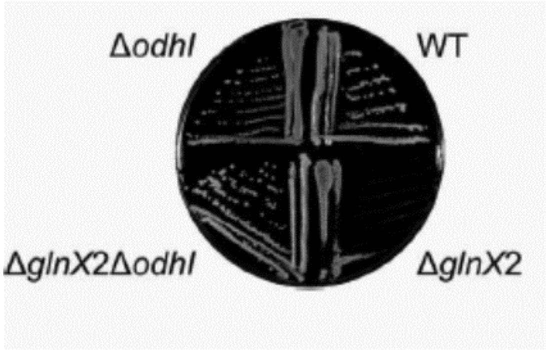

Fig. S3. Growth of the indicated *C. glutamicum* strains on agar plates with L-glutamine as sole carbon and nitrogen source.

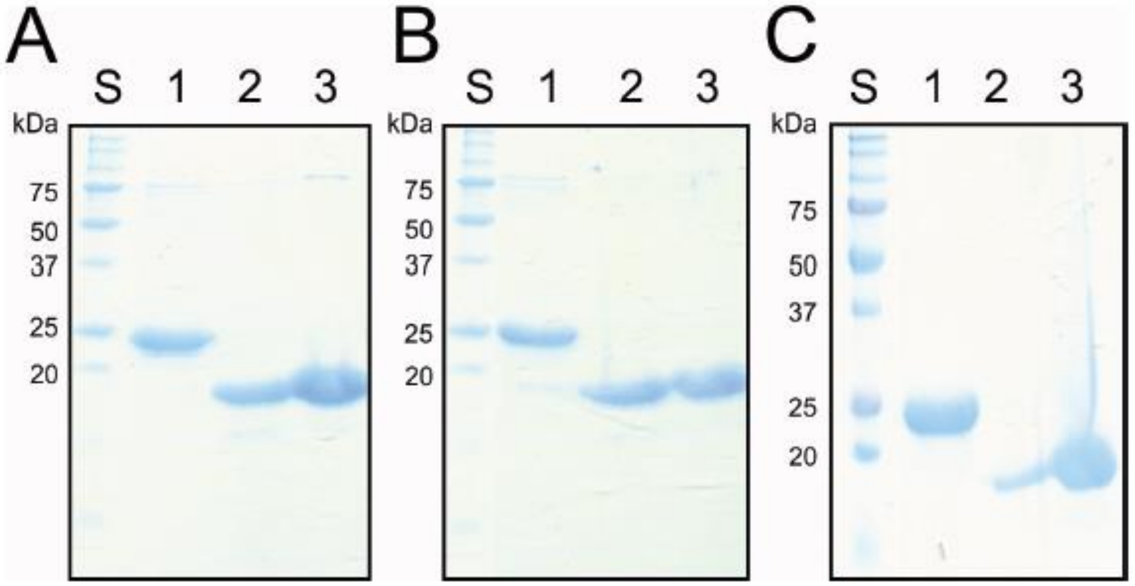

Fig. S4. Purification of OdhI (A), OdhI-R87A (B) and OdhI-R87P (C). The proteins were overproduced in *E. coli* BL21(DE3) using the expression plasmids pET-TEV-odhI, pET-TEV-odhI-R87P und pET-TEV-odhI-R87A and purified by Ni-NTA affinity chromatography (lane 1). Subsequently, the N-terminal His-tag was cleaved off by TEV protease (lane 2) and the buffer was exchanged (lane 3) against buffer B (0.1 M TES/NaOH, pH 7.2; 10 mM $MgCl_2$; 3 mM cysteine; 30% (w/v) glycerol) using PD10 columns for subsequent ODH activity assays. The protein samples were mixed with 6 x SDS loading buffer and analyzed by SDS-PAGE and Coomassie staining.

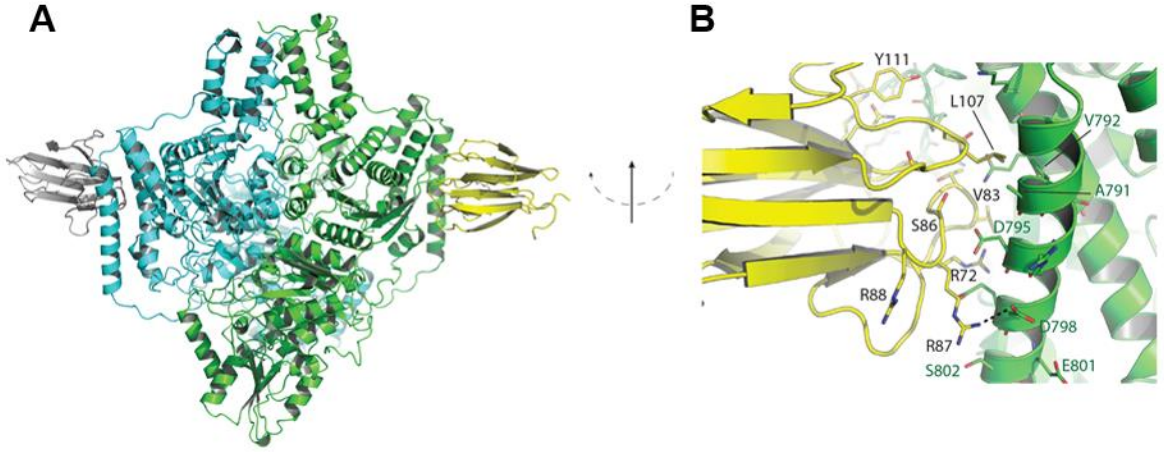

Fig. S5. (a) Cartoon representation of the AlphaFold2 model for the OdhA:OdhI complex, limited to the OdhA E1o domain. The model was generated as a 1:1 complex, and the OdhA E1o dimer was reconstructed by superimposition to the dimeric $KGD_{\Delta 360}$:GarA 2:2 complex (PDB 6I2Q). (b) Zoomed view of the OdhA:OdhI interaction, focusing on the OdhA αE helix.

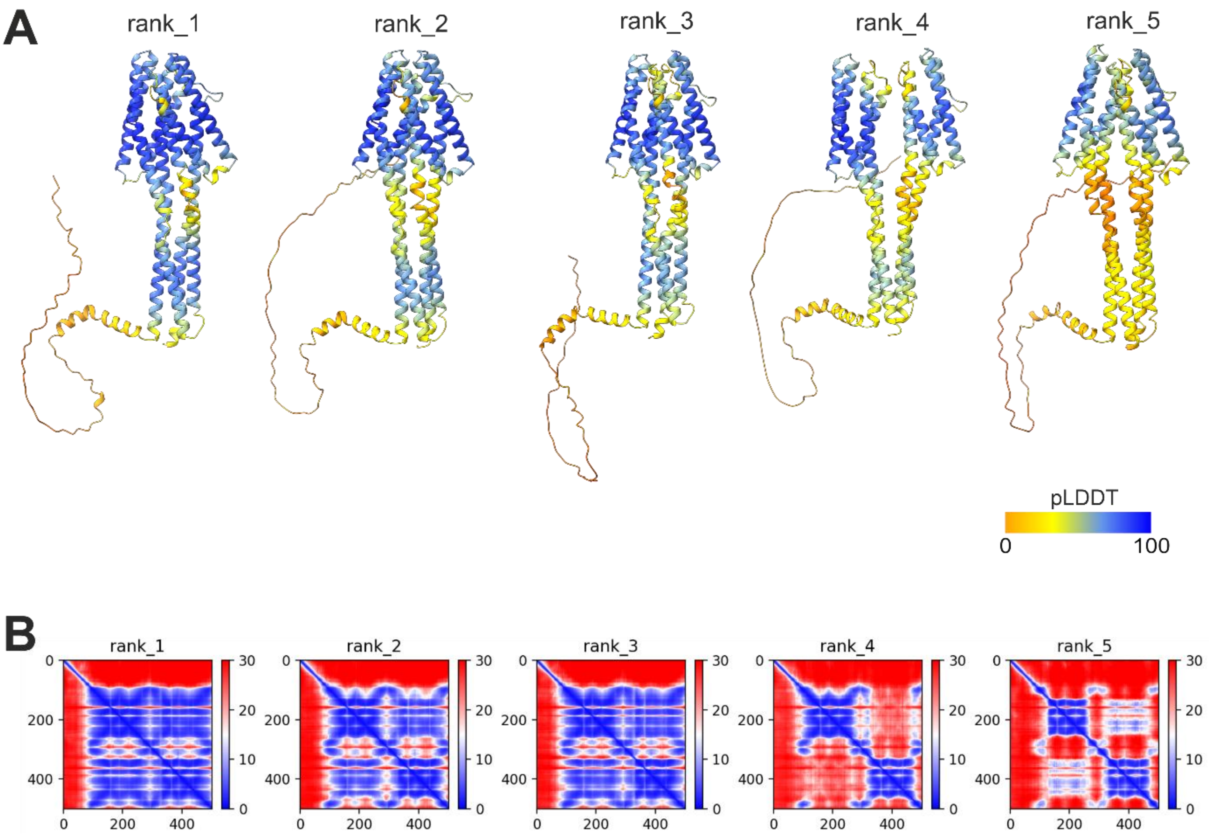

Fig. S6. Individual GlnX models predicted by ColabFold, powered by AlphaFold2. A. The five predicted models for GlnX are coloured according to their respective pLDDT scores. B. Heat maps of PAE (predicted aligned error) values for each model depicting the per-residue position confidence (adopted from ColabFold run output). The overall confidence of the models is based on both pLDDT and PAE values.

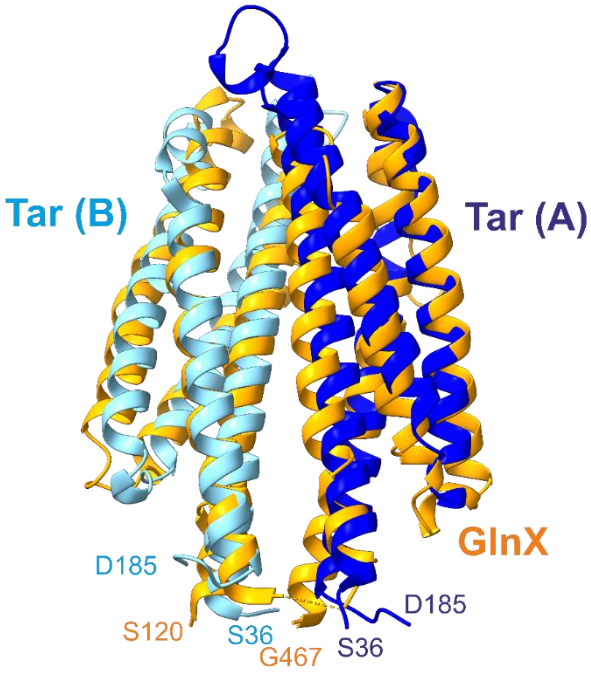

Fig. S7. Superimposition of GlnX with the Tar chemoreceptor. The predicted GlnX periplasmic tandem 4HB (orange) and the dimeric periplasmic domain of Tar from *Escherichia coli* (chain A, blue; chain B, cyan; PDB code 4Z9J) share a similar overall architecture.

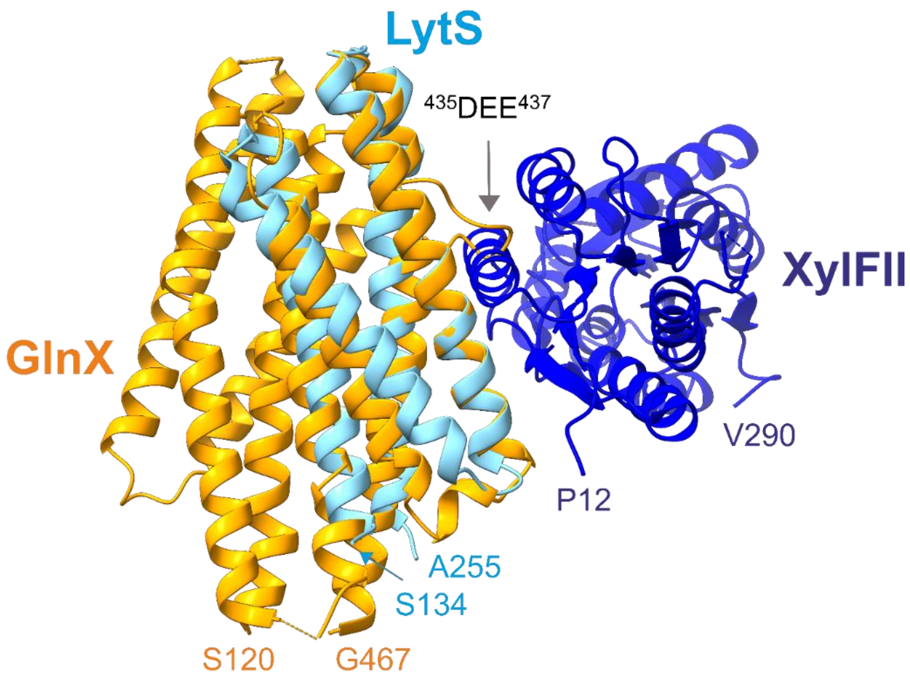

Fig. S8. Superimposition of GlnX with the LytS-XylFII complex (PDB code 5XSJ). Alignment of LytS (cyan) with the second 4HB module of GlnX (orange) places the periplasmic binding protein XylFII (blue) close to the acidic protrusion of GlnX helix H4'.
